# DNA based neoepitope vaccination induces tumor control in syngeneic mouse models

**DOI:** 10.1038/s41541-023-00671-5

**Published:** 2023-05-27

**Authors:** Nadia Viborg, Michail Angelos Pavlidis, Marina Barrio-Calvo, Stine Friis, Thomas Trolle, Anders Bundgaard Sørensen, Christian Bahne Thygesen, Søren Vester Kofoed, Daniela Kleine-Kohlbrecher, Sine Reker Hadrup, Birgitte Rønø

**Affiliations:** 1 Evaxion Biotech, Hørsholm, Denmark; 2 Department of Health Technology, Technical University of Denmark, Lyngby, Denmark

**Keywords:** DNA vaccines, Immunosurveillance, Immunosurveillance

## Abstract

Recent findings have positioned tumor mutation-derived neoepitopes as attractive targets for cancer immunotherapy. Cancer vaccines that deliver neoepitopes via various vaccine formulations have demonstrated promising preliminary results in patients and animal models. In the presented work, we assessed the ability of plasmid DNA to confer neoepitope immunogenicity and anti-tumor effect in two murine syngeneic cancer models. We demonstrated that neoepitope DNA vaccination led to anti-tumor immunity in the CT26 and B16F10 tumor models, with the long-lasting presence of neoepitope-specific T-cell responses in blood, spleen, and tumors after immunization. We further observed that engagement of both the CD4+ and CD8+ T cell compartments was essential to hamper tumor growth. Additionally, combination therapy with immune checkpoint inhibition provided an additive effect, superior to either monotherapy. DNA vaccination offers a versatile platform that allows the encoding of multiple neoepitopes in a single formulation and is thus a feasible strategy for personalized immunotherapy via neoepitope vaccination.

## Introduction

T cells are acknowledged to play an essential role in the immunological recognition and rejection of tumors^1^. Mutation-derived T-cell epitopes, also known as neoepitopes, are actively explored as therapeutic cancer targets as they differentiate aberrant tumor cells from the normal healthy cells in the body. Neoepitope-specific T cells have been reported in peripheral blood and tumor of patients with various solid cancers^2–6^. Furthermore, the advent and approval of checkpoint inhibitor (CPI) therapy has transformed the field of cancer immunotherapy to become the standard of care (SoC) in several disease indications. Durable responses to CPI therapy and associated favorable cancer prognosis have been related to tumor mutational burden or neoepitope load and the intratumoral presence of T cells, while neoepitopes have shown to be a prime target for immune responses raised upon CPI therapy^7–12^.

With tumor-restricted expression, neoepitopes are considered ideal therapeutic cancer targets that are minimally affected by immune tolerance and harbor a limited risk of autoimmune adverse events. Therapeutic cancer vaccination with patient-specific neoepitopes offers a promising strategy to harness immune responses against a tumor. Such personalized strategies have recently been pursued in early clinical trials with encouraging results when delivering the patient-specific neoepitopes loaded on autologous dendritic cells^13^, encoded by RNA^14,15^ or as neopeptide pools adjuvanted by polyinosinic–polycytidylic acid complexed with poly-l-lysine (poly-ICLC)^16,17^. In each of these clinical trials, several neoepitopes were able to enhance existing or induce de novo T-cell responses, confirming the immunogenicity of neoepitopes in humans. Interestingly, the majority of the T-cell responses observed in these clinical trials with RNA- and peptide-based delivery were major histocompatibility complex (MHC) class II-restricted and thus recognized by CD4+ T cells. A multitude of ongoing and completed clinical trials assess patient-tailored neoepitope vaccines in combination with SoC CPI therapy. These combination therapies hold great promise and have the potential for a dual strike against the tumor where CPI therapy can release the ‘brakes’ imposed on the immune system, which is then accompanied by specifically ‘steering’ the new and/or amplified immune responses towards the tumor cells.

Preclinical research investigating neoepitope-targeting immunotherapy constitutes a fast-growing field. Several neoepitopes identified in a range of mouse tumor models, such as CT26^18^, MC38^19,20^, B16F10^18,21,22^, and GL261^22^ have been employed in vaccine interventions with marked therapeutic results. These murine tumors originate from different tissue types and, once transplanted to inbred mice, they manifest diverse levels of immunogenicity and sensitivity to immunotherapy^23^. Various vaccine platforms, including synthetic neopeptides adjuvanted by the Toll-like receptor (TLR) agonists polyinosinic–polycytidylic acid (poly IC) or CpG, are capable of inducing tumor growth delay or eradication in MC38 and B16F10 tumor models^19,20,24^. Similarly, preclinical studies with self-adjuvanting, neoepitope-encoding mRNA^18,25^, or self-replicating RNA^26^ demonstrated the immunogenicity, safety, and therapeutic relevance of this approach.

DNA vaccination harbors self-adjuvating properties and stimulates the innate DNA sensing machinery of mammalian cells. This directs the immune response towards Th1-like immunity, which has shown favorable in effective immune responses towards cancer, e.g. via Th1-prototypical cytokine interferon (IFN)γ^27–29^. Antigens delivered in a DNA format have direct access to the MHC class I processing and presentation pathway in transfected cells, which facilitates the induction of cytotoxic CD8+ T cells. Delivery systems and adjuvants are commonly employed to facilitate efficient vaccination with various antigen formats. For DNA vaccination, delivery systems can protect against potential degradation of the DNA plasmid after administration and increase transfection efficiency^30^. Nonionic block co-polymers form micelle-like structures with DNA and enhance gene delivery to several tissues^31,32^. Block co-polymers are hypothesized to add adjuvant functions that augment the immunogenicity of DNA via directing immune responses on the Th1/Th2 axis based on their chemical properties^33^.

In this study, we set out to investigate the immunogenicity and anti-tumor efficacy of in silico-predicted neoepitopes delivered as plasmid DNA in the CT26 and B16F10 tumor models. Through our observations, we wish to increase our understanding of the T-cell immune response that supports the ability to prevent or reject tumor growth following neoepitope-targeting immunotherapy.

## Results

### Prophylactic immunization with CT26 neoepitopes delivered as plasmid DNA inhibits tumor growth and induces neoepitope-recognizing T cells

**Fig. 1:**
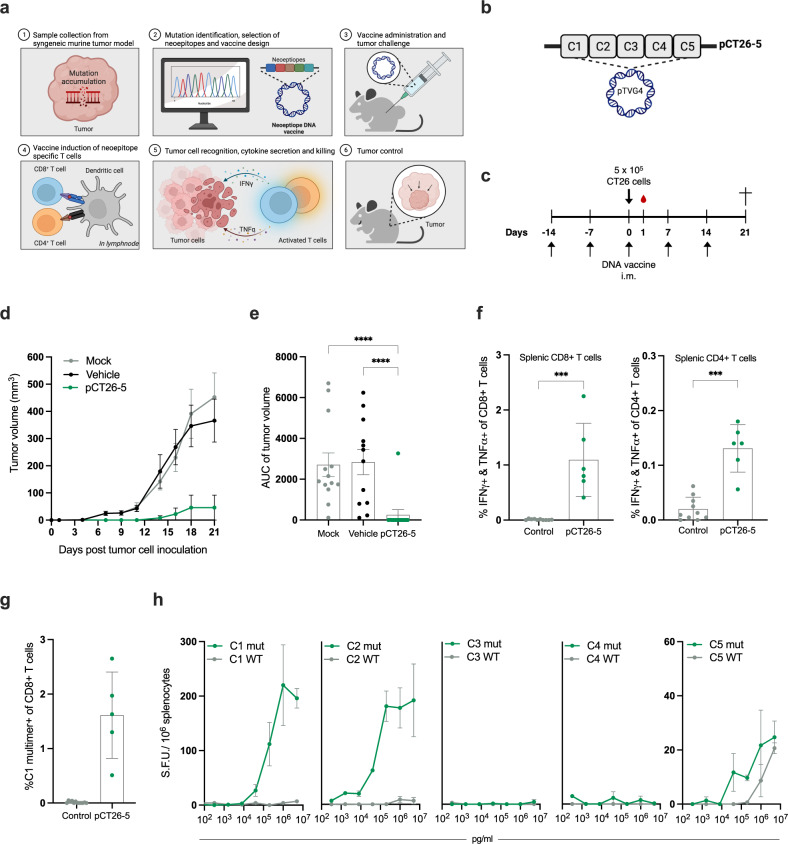
In vivo and ex vivo efficacy of vaccination with neoepitope encoding DNA. **a** Visualization of our approach to identify, select and assess tumor neoepitopes in preclinical models. **b** Schematic of the pCT26-5 DNA plasmid with the five-neoepitope insert. **c** Representation of the timeline in the in vivo experiment. Groups of *n* = 12–13 BALB/c mice were immunized prophylactically with 100 µg of pCT26-5 DNA, Mock DNA, or vehicle, before s.c. inoculation with CT26 tumor cells. Naïve control mice (no immunizations and no tumor inoculation) were housed together with experimental mice in a mixed cage setup. **d** Group mean tumor growth curves (in mm^3^) ± standard error of the mean (SEM). **e** Area under the tumor growth curve (AUC) for individual mice by group ± SEM. **f** Peptide pool re-stimulation and intracellular cytokine staining for interferon (IFN)γ and tumor necrosis factor (TNF)α producing CD4+ and CD8+ T cells on bulk splenocytes (*n* = 3–6 mice per group, control: mock, vehicle and naïve, mean ± standard deviation, SD). **g** Tail vein blood collected in EDTA-coated tubes was stained on study day 1 with an MHC multimer (loaded with H-2K^d^ restricted minimal epitope *KFKASRASI* from the C1 neoepitope), to monitor the frequency of antigen-specific CD8+ T cells induced by immunization (*n* = 3-6 mice per group, mean ± SD). **h** IFNγ ELISpot on splenocytes upon re-stimulation with immunization-relevant neoepitope or wild-type (WT) sequences (in technical triplicates, mean ± SD). *Statistics*: Kruskal–Wallis test with Dunn’s multiple comparisons correction (**e**) and Mann–Whitney test (**f** and **g**), *p < 0.05, **p < 0.01, ***p < 0.001, ****p < 0.0001.

We initiated our analyses in the BALB/c syngeneic colon carcinoma model CT26 which has previously been investigated for its high neoepitope load^34^ and relevance in immunotherapeutic interventions^23^. Parallel next-generation sequencing and analysis of DNA and RNA from the CT26 tumor cell line and healthy tissue DNA facilitated the mapping of nonsynonymous somatic mutations to the CT26 tumor cell line (Fig. 1a). This process led to artificial intelligence (AI)- guided selection of the top-ranked neoepitopes for immunization with the following characteristics: (1) a 27mer amino acid (AA) sequence with the somatic mutation in the center position flanked by the wild type (WT) sequences, (2) each neoepitope is validated for expression via RNA sequencing, and 3) the neoepitopes are prioritized to represent the top five in silico predicted MHC class II ligands (H-2-IA^d^). Subsequent in silico analysis of the top-ranked neoepitopes showed that they each contain a predicted strong binder for H-2^d^ class I alleles in addition to the class II binder, thus potentially able to induce both CD4+ and CD8+ T-cell responses as is reported to be essential in murine models of cancer immunotherapy (Supplementary Table 1)^18,35^.

pTVG4 plasmid DNA encoding the top five selected CT26 neoepitope sequences C1–C5 (Supplementary Table 1, from here on: pCT26-5, Fig. 1b) was formulated with nonionic block co-polymer (from here on: poloxamer) which has been described to function as adjuvant and furthermore facilitate DNA delivery to cells and thereby increase antigen expression and immune stimulation^33^. In a prophylactic setup, we evaluated anti-tumor effect and immunogenicity of intramuscularly (i.m.) immunized BALB/c mice upon dosing with either pCT26-5, empty plasmid without neoepitope payload (‘mock’), or poloxamer only (‘vehicle’). All mice received weekly immunizations over the course of the experiment. Two weeks after priming, mice were inoculated with CT26 tumor cells subcutaneously (s.c.) in the right flank (Fig. 1c). Mice immunized prophylactically with pCT26-5 developed significantly smaller or no tumors compared to mock DNA or vehicle-treated mice (Fig. 1d and e) with a total of 12 out of 13 complete responders in the pCT26-5 group. We did also attempt to immunize mice with pCT26-5 after tumor inoculation (therapeutically) but did not observe control of CT26 tumors after the challenge (Supplementary Fig. 1). To explore the immune response induced by pCT26-5 immunization, we first applied the MHC class I multimer (MHC multimer) staining methodology. We used MHC multimers loaded with a minimal epitope (KFKASRASI) derived from the C1 neoepitope to stain tail vein blood.

Two weeks after priming immunization, we observed a significantly higher frequency of C1-specific CD8+ T cells in mice immunized with pCT26-5 than in control mice (Fig. 1g). Epitope recognition is an important step towards anti-tumor immunity, however, to induce cancer cell killing a functional immune response is required. To investigate the functional T-cell responses induced by neoepitope immunization, splenocytes were re-stimulated with neopeptides corresponding to the pCT26-5 vaccine cargo, and cytokine-producing CD4+ and CD8+ T cells were subsequently detected by flow cytometry. pCT26-5 immunization resulted in high frequencies of double-cytokine producing CD8+ T cells and CD4+ T cells upon neopeptide ex vivo stimulation (Fig. 1f). We conducted a similar immune analysis in the study with a comparison of prophylactic and therapeutic pCT26-5 immunization, where we observed a tendency of higher frequencies of specific CD4+ and CD8+ T cells resulting from prophylactic immunization (Supplementary Fig. 1). Collectively, these data demonstrate that the potent anti-tumor effect is accompanied by an induction of antigen-specific T cells after neoepitope DNA vaccination.

To investigate if neoepitope immunization elicits immune reactivity to the WT sequences present in healthy cells, we used the neopeptides of pCT26-5 and their WT counterparts to re-stimulate splenocytes and assess reactivity. Via IFNγ ELISpot, we observed that three neoepitopes from pCT26-5 were immunogenic (C1, C2, and C5) and confirmed the preferential recognition of the mutated over the WT peptide sequences (Fig. 1h). The WT counterpart of C5 neoepitope was recognized from 10^6^ pM stimulation concentration, albeit resulting in lower spot forming units (SFUs) than the C5 neoepitope. These observations align well with descriptions in the literature underlining that cross-reactivity to corresponding WT sequence after immunization with tumor neoepitopes is rare^16,36^.

### CD4+ and CD8+ T cells induced by neoepitope DNA are instrumental to control tumor growth

**Fig. 2:**
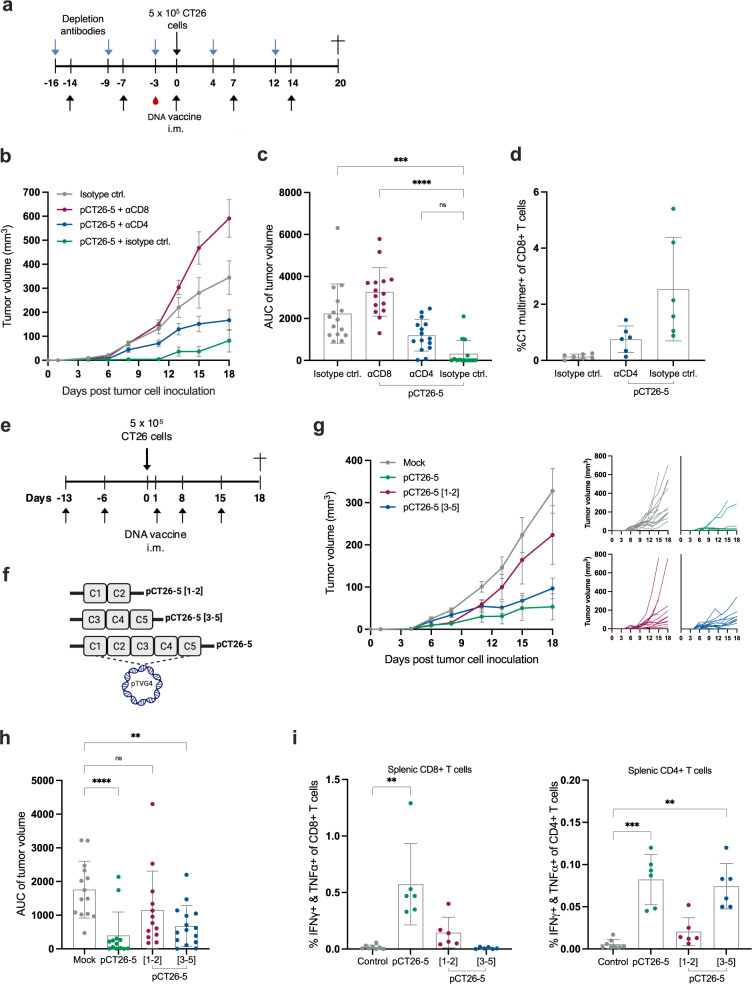
CD4+ and CD8+ T cells partake in anti-tumor capacity from neoepitope DNA immunization. **a** Representation of the timeline in the first in vivo experiment. Groups of *n* = 14–15 BALB/c mice were administered antibodies to selectively deplete CD4+ or CD8+ T cells (or isotype control antibody) via i.p. administration, and concurrently immunized prophylactically with 50 µg of pCT26-5 DNA before s.c. inoculation with CT26 tumor cells. **b** Group mean tumor growth curves (in mm^3^) ± SEM of in vivo experiment (**a**). **c** Tumor volume AUC for individual mice by a group of in vivo experiment (**a**), mean ± SEM. **d** Tail vein blood collected in EDTA-coated tubes was stained on study day-3 with neoepitope C1-MHC multimer to monitor the frequency of antigen-specific CD8+ T cells induced by immunization of in vivo experiment (**a**), *n* = 5–6 mice per group, mean ± SD. **e** Representation of the timeline in the second in vivo experiment. Groups of *n* = 13–14 BALB/c mice were immunized prophylactically with 50 µg of DNA vaccine before s.c. inoculation with CT26 tumor cells. Naïve control mice (no immunizations and no tumor inoculation) were housed together with experimental mice. **f** Schematic of the DNA plasmids with the neoepitope subset inserts. **g** Left: Group means tumor growth curves (in mm^3^) ± SEM; right: individual tumor growth curves; of in vivo experiment (**e**). **h** Tumor volume AUC for individual mice by a group of in vivo experiment (**e**), mean ± SEM. **i** Peptide pool re-stimulation and intracellular cytokine staining for IFNγ and TNFα producing CD4+ (left) and CD8+ (right) T cells on bulk splenocytes (*n* = 4–6 mice per group, control: mock and naïve splenocytes) of in vivo experiment (**e**), mean ± SD. *Statistics*: Kruskal–Wallis test with Dunn’s multiple comparisons correction (**c**, **h**, and **i**), *p < 0.05, **p < 0.01, ***p < 0.001, ****p < 0.0001.

To explore the immune response that is associated with CT26 tumor rejection, we combined prophylactic pCT26-5 DNA vaccination with anti-(α)CD4 or αCD8 T-cell depleting antibodies, to delineate the extent to which the observed tumor control is reliant on either T-cell subset (Fig. 2a). Depletion of CD8+ T cells completely abrogated the ability of pCT26-5 to prevent tumor growth, which underlines the essential role of CD8+ T cells in mediating a tumoricidal immune response (Fig. 2b and c). The effect from the depletion of CD4+ T cells was less detrimental, as the majority of these mice still developed tumors, albeit at lower average volumes than the non-vaccinated isotype control mice. Via MHC multimer staining, we observed that there were less C1 neoepitope-recognizing CD8+ T cells in the blood of pCT26-5 immunized mice whose CD4+ T cells had been selectively depleted (Fig. 2d). Hence the suggested role of CD4+ T-cells is to shape and improve the CD8+ T-cell response that is induced, though CD4+ T cells do not restrain the tumor growth as much as CD8+ T cells. In this experiment, the administration of depletion antibodies was initiated prior to neoepitope vaccination, and hence neither the T-cell subset was able to partake in shaping the immune response nor to facilitate direct or indirect tumor rejection. In a separate experiment where T-cell subsets were depleted two weeks later, i.e. simultaneously with tumor cell inoculation, hence allowing the pCT26-5 immune response to initiate before depletion, we observed again how CD8+ T cells were required to obtain tumor control (Supplementary Fig. 2). Meanwhile, mice who were immunized with pCT26-5 and then late depleted of their CD4+ T cells in this experiment showed the similar capacity to control tumor growth as pCT26-5 non-depleted (isotype control) mice. These observations underline that CD4+ T cells play a role in shaping and priming the immune response but are not as crucial to facilitate direct tumor cell killing as the CD8+ T cells.

To further assess how the enclosed pCT26-5 neoepitopes contribute or cooperate towards tumor control and immunogenicity, we designed two new DNA plasmids with subsets of the aforementioned neoepitopes: pCT26-5 [1–2] encoding neoepitopes C1 and C2, and pCT26-5 [3–5] encoding neoepitopes C3, C4, and C5 (Fig. 2f) and explored the anti-tumor effect of each neoepitope subset plasmid by immunizing mice inoculated with CT26 tumor cells (Fig. 2e). From this study it was apparent that the subgroup plasmids conferred only partial tumor control relative to pCT26-5 (Fig. 2g and h). A comparison of the induced T-cell responses in splenocytes showed that while the full pCT26-5 induced strong neoepitope recognition within both CD4+ and CD8+ T cells, pCT26-5 [1–2] subset resulted mainly in a lower frequency of specific CD8+ T cells, and pCT26-5 [3–5] led exclusively to specific CD4+ T cell recognition (Fig. 2i).

Together, these data indicate that both CD4+ and CD8+ T-cell subsets take part in mediating the anti-tumor effect and shaping the immune response of neoepitope DNA vaccination. This is evidenced by how the neoepitope-specific CD8+ T-cell response is hampered when CD4+ T-cell help is lacking; either from CD4+ T cells as a whole or from neoepitope-specific CD4+ T cells.

### An increased neoepitope number and optimized DNA construct design result in complete protection from tumor challenge

**Fig. 3:**
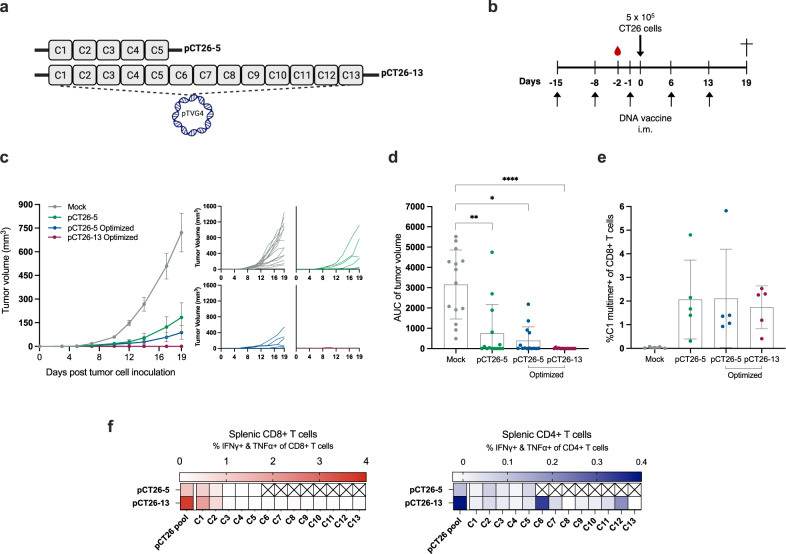
Higher number of DNA-encoded neoepitopes confers complete prevention of tumor growth and a broad immune response. **a** Schematic of the DNA plasmids with 5 or 13 neoepitopes. Groups of *n* = 13–14 BALB/c mice were immunized prophylactically with 50 µg of pCT26-5 (either the non-optimized or sequence optimized plasmid, ‘Optimized’) or pCT26-13-Optimized DNA plasmid before s.c. inoculation with CT26 tumor cells. **b** Representation of the timeline in the in vivo experiment. **c** Left: Group mean tumor growth curves (in mm^3^) ± SEM, right: tumor growth of individual mice. **d** Tumor volume AUC for individual mice by group, mean ± SEM. **e** Tail vein blood collected in EDTA-coated tubes was stained on study day −2 with neoepitope C1-MHC multimer to monitor the frequency of antigen-specific CD8+ T cells induced by immunization, mean ± SD. **f** Peptide pool or individual peptide re-stimulation and intracellular cytokine staining for IFNγ and TNFα producing CD4+ and CD8+ T cells on bulk splenocytes, visualized by heatmap (*n* = 2–4 mice per group). *Statistics*: Kruskal–Wallis test with Dunn’s multiple comparisons correction (**d**). *p < 0.05, **p < 0.01, ***p < 0.001, ****p < 0.0001.

Multiple reports from the literature have showcased that merely a fraction of screened neoepitopes in mice and humans tend to be immunogenic, with even fewer neoepitopes associated with tumor rejection upon immunotherapy. For this reason and to steer T cells towards multiple neoepitope targets for potentially more potent tumor recognition, we investigated if including more neoepitopes in the DNA plasmid could facilitate enhanced efficacy, why we inserted and tested *n* = 13 neoepitopes. These were selected as described previously for the pCT26-5 but expanded to include the top 13 predicted CT26 neoepitopes (from here on: pCT26-13, Supplementary Table 1, Fig. 3a). As our DNA construct consists of neoepitopes randomly concatenated and separated by sequences encoding the same glycine and serine linkers, manufacturing may prove troublesome due to repeats, poor GC content or unwanted sequence features. To address this, we applied an in-house developed bioinformatic tool that optimizes DNA plasmid sequences prior to synthesis for codon optimization, GC content, repeats, TATA boxes, secondary RNA structures, and premature poly-A tails. This tool ensures that the DNA plasmids can be manufactured and potentially increases the expression of DNA, which is of particular importance in the setting of personalized vaccines where there is a need to incorporate patient-tailored payloads, and where suboptimal DNA designs can lead to long production times. In the described experiment we observed that prophylactic immunization with 50 μg of the optimized pCT26-13 prevented CT26 tumors from arising in all mice, while a fraction of mice immunized with 50 μg of the non-optimized pCT26-5 plasmid developed tumors, in agreement with previous experiments (Fig. 3c and d). The non-optimized and optimized DNA sequence of the pCT26-5 construct performed comparably, resulting in the same number of tumor-bearing mice at study termination. MHC multimer staining showed similar levels of C1 neoepitope-specific CD8+ T cells in peripheral blood for all groups (Fig. 3e). Via ICS analyses of the splenic T-cell compartment, we observed distinct profiles of neoepitope recognition resulting from pCT26-5 and pCT26-13 immunization (Fig. 3f). CD8+ T-cell reactivity was exclusive to neoepitopes C1 and C2 for both plasmids. The CD4+ T-cell responses were distributed as follows; pCT26-5 with primarily C2 and C5 reactivity, and pCT26-13 with C2, C6, and C12 reactivity, showing how the inclusion of more neoepitopes resulted in a broader T-cell response and recognition of more neoepitopes.

In parallel, we turned to investigate whether the observed neoepitope immune responses were resulting from the neoepitope vaccines or could be attributed to the tumor challenge. To address that question, groups of mice were prophylactically vaccinated with 50 µg of optimized pCT26-13 or mock DNA and subsequently challenged with CT26 cells (Supplementary Fig. 3). The mice were interrogated for CD8+ T-cell recognition of the C1 neoepitope specificity via MHC multimer staining in blood 13 days post-tumor inoculation and in splenic cell suspensions generated after sacrifice of mice on day 21. We observed that C1 neoepitope was recognized by T cells in blood and spleens of the pCT26-13 group, whereas no vaccine-neoepitope immunity was detected in the mock DNA group or naïve mice (Supplementary Fig. 3). Finally, to investigate whether functional responses were elicited against the vaccine-encoded neoepitopes, splenocytes were assayed in IFNγ ELISpot after stimulation with the vaccine-neoepitope pool. This analysis confirmed that mice injected with mock DNA and naïve mice show no reactivity against the neoepitope pool, whereas the pCT26-13 group harbored strong immune responses.

Together, the data support the inclusion of more neoepitopes for immunization to achieve an immune response of increased broadness and magnitude, which might explain the superior ability of pCT26-13 to prevent tumor growth. Based on these results, the sequence optimized pCT26-13 was used for all subsequent experiments in the CT26 model, simply denoted as pCT26-13, and the optimization tool demonstrated the ability to generate DNA sequences capable of eliciting functional immune responses and capacity to hamper tumor growth.

### Checkpoint inhibitor combination therapy improves efficacy of sub-optimal dose neoepitope DNA immunization

**Fig. 4:**
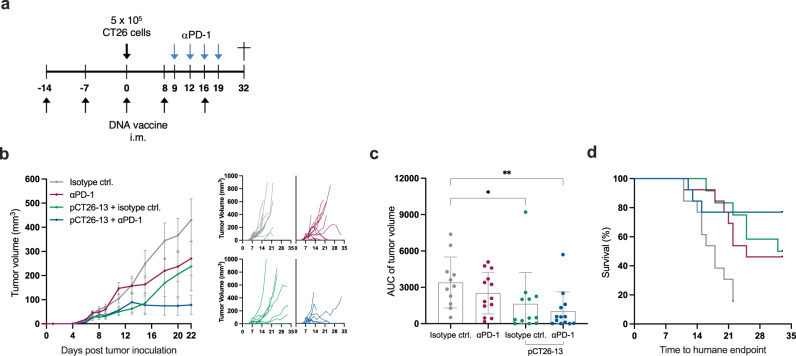
Additive effect of low-dose neoepitope DNA immunization and αPD-1 combination therapy. **a** Representation of the timeline in the in vivo experiment. Groups of *n* = 11–13 BALB/c mice were immunized prophylactically with 3 µg of pCT26-13 DNA before s.c. inoculation with CT26 tumor cells. Administration of αPD-1 or isotype control antibody was initiated when untreated control tumors reached an average volume of approximately 100 mm^3^. **b** Left: Group mean tumor growth curves (in mm^3^) ± SEM until day 22 when the majority of the mice were still alive; right: tumor growth of individual mice for the duration of the study. **c** Tumor volume AUC for individual mice by group, mean ± SEM. **d** Kaplan–Meier plot of survival for the duration of the study. *Statistics*: Kruskal–Wallis test with Dunn’s multiple comparisons correction (**c**), *p < 0.05, **p < 0.01.

Next, we investigated the potential of combining neoepitope DNA vaccination with an anti-PD-1 (αPD-1) immune checkpoint inhibitor, a current standard of care for several solid cancer indications. Since we observed that a 50 μg dose of pCT26-13 prevented tumor growth in all mice, we applied a sub-optimal (non-protective) dose of 3 μg pCT26-13 DNA allowing a window for the therapeutic effect of the combination therapy (Fig. 4a). The study showed an additive effect of the pCT26-13 and αPD-1 combination therapy in reducing the tumor burden compared to each monotherapy on their own (Fig. 4b and c) as well as prolonged survival, marked as time to reach humane endpoint (Fig. 4d). This combination therapy mimics an ongoing clinical phase 1/2 trial (*NCT04455503*), in which fully resected melanoma patients are treated with αPD-1 (Nivolumab) and eight i.m. doses of personalized neoepitope DNA plasmid as adjuvant immunotherapy. Each DNA plasmid encodes 13 patient-tailored neoepitopes and is subjected to bioinformatical sequence optimization prior to synthesis, as described in the above paragraph, hence the pCT26-13 represents a murine surrogate for the clinical personalized plasmid compounds.

**Fig. 5:**
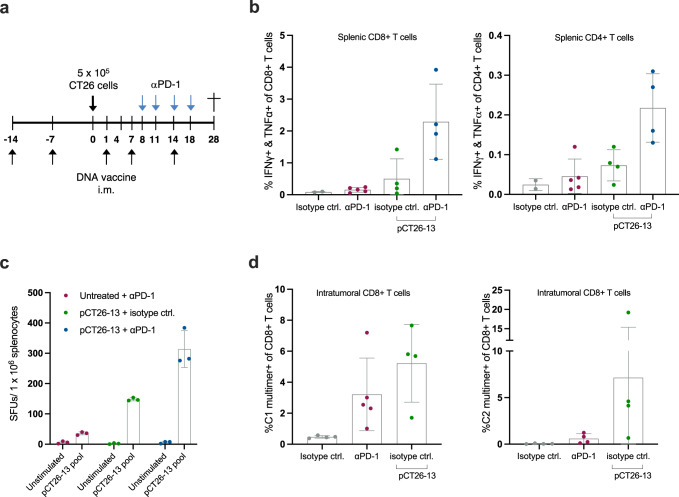
Combination therapy induces favorable immunogenicity compared to monotherapies. **a** Representation of the timeline in the in vivo experiment. Groups of *n* = 13–15 BALB/c mice were immunized prophylactically with 3 µg of pCT26-13 DNA before s.c. inoculation with CT26 tumor cells. Administration of αPD-1 or isotype control antibody was initiated when untreated control tumors reached an average volume of approximately 100 mm^3^. Naïve control mice (no immunizations and no tumor inoculation) were housed together with experimental mice in a mixed cage setup. **b** Peptide pool re-stimulation and intracellular cytokine staining for IFNγ and TNFα producing CD4+ and CD8+ T cells on bulk splenocytes (*n* = 2–5 mice per group, mean ± SD). **c** Peptide pool re-stimulation and IFNγ ELISpot on splenocytes (*n* = 3 mice per group, mean ± SD). **d** Tumor digests single cell suspensions stained after termination with neoepitope C1 and C2-MHC multimers to monitor the frequency of antigen-specific CD8+ T cells (*n* = 4–5 mice per group, mean ± SD).

In a separate pCT26-13 and αPD-1 combination experiment (Fig. 5a), we investigated the accompanying immune responses. Within splenocytes we found a higher frequency of neoepitope-responsive CD4+ and CD8+ T-cells from the combination therapy than pCT26-13 monotherapy via ICS (Fig. 5b) and IFNγ ELISpot (Fig. 5c). From the ELISpot analysis it was also evident that αPD-1 monotherapy induced a feeble immune recognition within splenocytes towards the plasmid-encoded neoepitopes despite not being actively immunized against them. Tumor single cell suspensions were subjected to MHC multimer staining to detect tumor infiltrating CD8+ T-cells specific to neoepitopes C1 and C2 (the combination therapy resulted in no or small tumors, hence no tumor cell suspensions were available for this analysis). pCT26-13 monotherapy induced C1 and C2 neoepitope specific CD8+ T-cells in tumors to a comparable frequency (Fig. 5d).

Interestingly, C1 and C2 neoepitope immune recognition also arose upon αPD-1 monotherapy. Hence, our results show the presence of T-cells specific to plasmid-encoded neoepitopes in several compartments.

### Neoepitope DNA immunization results in durable immune responses and protection from late CT26 tumor challenge

**Fig. 6:**
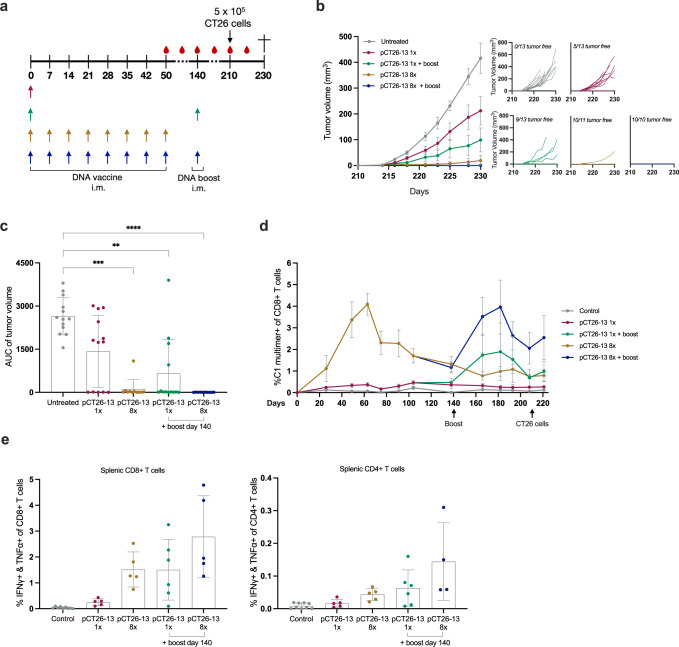
pCT26-13 confers long-term protection from tumor challenge and a durable immune response. **a** Representation of the timeline in the in vivo experiment. Groups of *n* = 10–13 BALB/c mice were immunized from day 0 with 1× or 8× pCT26-13 immunizations (25 µg per dose), with and without late booster immunization on day 140, and subsequent CT26 tumor challenge on day 210. **b** Left: Group means tumor growth curves (in mm^3^) ± SEM; Right: tumor growth of individual mice. **c** Tumor volume AUC for individual mice by group, mean ± SD. **d** Tail vein blood was collected in EDTA-coated tubes throughout the study and stained with neoepitope C1-MHC multimer to monitor the frequency of antigen-specific CD8+ T cells induced by immunization (*n* = 3–6 mice per group, control: blood from age matching and co-housed-naïve controls, mean ± SD). **e** Peptide pool re-stimulation and intracellular cytokine staining for IFNγ and TNFα producing CD4+ and CD8+ T cells on bulk splenocytes (*n* = 4–6 mice per group, control: untreated and naïve splenocytes, mean ± SD). *Statistics*: Kruskal–Wallis test with Dunn’s multiple comparisons correction (*D*), *p < 0.05, **p < 0.01, ***p < 0.001, ****p < 0.0001.

Next, we set out to assess the longitudinal effects of neoepitope DNA immunization regarding immunogenicity and tumor control. We designed an experiment with a varying immunization schedule of 1 (1×) or 8 (8×) pCT26-13 immunizations including a late booster immunization 140 days after priming for specified groups (Fig. 6a). Mice were subsequently challenged with CT26 tumors 210 days after the first immunization. We observed that untreated control mice developed tumors after the challenge while pCT26-13 dosing led to varying degrees of tumor control (Fig. 6b and c). 8× immunizations ±boost on day 140 prevented tumor growth in all but one mouse, hence in these groups the tumor control from pCT26-13 immunization was comparable to prior experiments that employed a much earlier tumor challenge. Interestingly, 1× immunization on day 0 with pCT26-13 DNA conferred tumor prevention in 5/13 mice, while there was a convincing additive effect of 1× + booster immunization schedule resulting in 9/13 tumor-free mice. The kinetics of the neoepitope immune response was continuously monitored during the experiment via C1 MHC multimer staining of peripheral blood (Fig. 6d). We observed the C1 neoepitope-specific CD8+ T-cell response to persist and increased in magnitude following late booster immunizations. Splenocytes harvested at the study endpoint allowed analyses of the functional T-cell responses via ICS, which indicated the presence of persevering neoepitope-specific CD4+ and CD8+ T-cells several months after the latest immunizations (Fig. 6e).

### Neoepitope DNA-mediated tumor control investigated in an additional syngeneic model

**Fig. 7:**
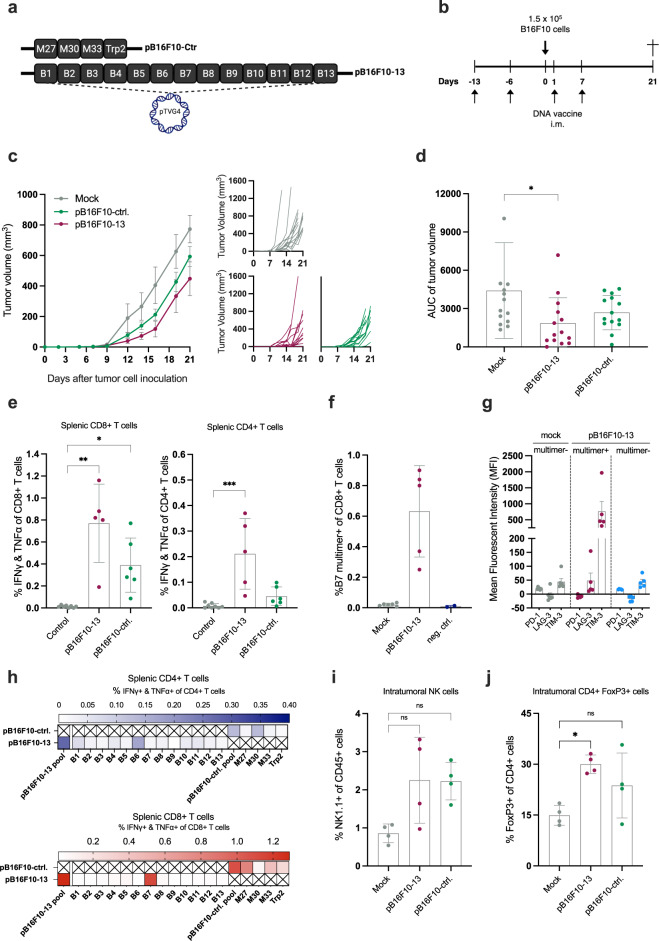
DNA encoding top 13 neoepitopes induces delay of tumor growth in an additional model. **a** Schematic of the DNA plasmids with 4 published epitopes (pB16F10-ctrl.) or 13 selected neoepitopes (pB16F10–13). **b** Representation of the timeline in the in vivo experiment Groups of *n* = 14 C57BL/6 mice were immunized prophylactically with 100 µg of pB16F10-13 or pB16F10-ctrl. (sequence-optimized plasmids) before s.c. inoculation with B16F10 tumor cells. **c** Left: Group means tumor growth curves (in mm^3^) ± SEM, right: tumor growth of individual mice. **d** Tumor volume AUC for individual mice by group, mean ± SEM. **e** Peptide pool re-stimulation and intracellular cytokine staining for IFNγ and TNFα producing CD4+ and CD8+ T cells on bulk splenocytes (*n* = 4-6 mice per group, control: mock and naïve splenocytes, mean ± SD). **f** Bulk splenocytes were co-stained with B7 MHC multimer comprising H-2Kb loaded with restricted minimal epitope SGFRYNVL from the B7 neoepitope and antibodies specific to PD-1, LAG-3, and TIM-3 surface markers to monitor the frequency of neoepitope-specific CD8+ T cells and the expression of exhaustion markers (*n* = 5–6 mice per group, mean ± SD). Two mice were stained with neg. ctrl. multimer (H-2K^b^ loaded with the restricted OVA-derived minimal epitope SIINFEKL). **g** Mean fluorescent intensity (MFI) for the three exhaustion markers within tetramer+ and tetramer- CD8+ T-cell populations defined in (**f**), mean ± SD. **h** Individual peptide re-stimulation and intracellular cytokine staining for IFNγ and TNFα producing CD4+ and CD8+ T cells on bulk splenocytes, visualized by heatmap (*n* = 2 mice IDs pooled per group to create one biological replicate). Single-cell suspensions of B16F10 tumor digests were phenotyped for % presence of (**i**) NK cells (NK1.1+) out of CD45+ cells and **j** FoxP3+ CD4+ cells (mean ± SD). *Statistics*: Kruskal–Wallis test with Dunn’s multiple comparisons correction (**d**, **e**, **f**, **i**, **j**), *p < 0.05, **p < 0.01, ***p < 0.001.

The ability of neoepitope DNA immunization to control tumor growth was investigated in the rapid-growing and immunologically cold B16F10 tumor model, syngeneic to C57BL/6 mice. We included two pTVG4-neoepitope encoding DNA plasmids in the experiment (Supplementary Table 2): pB16F10-13 (containing B16F10 predicted top 13 neoepitopes, AI-guided selection as described in the “Methods” section), and pB16F10-ctrl. (containing four literature-described B16F10 epitopes, including an epitope from melanoma differentiation antigen Trp2) (Fig. 7a and b). Both DNA plasmids were able to induce B16F10 tumor growth delay compared to mock DNA plasmid (Fig. 7c). However, only pB16F10-13 DNA immunization resulted in significantly lower tumor volume (Fig. 7c). The accompanying vaccine-specific immune response was assessed via peptide re-stimulation and ICS of splenocytes after study termination, where pB16F10-13 immunized mice displayed the highest levels of epitope-specific CD4+ and CD8+ T-cell responses (Fig. 7e), with several neoepitopes recognized by T cells (Fig. 7h). These results confirm the ability of DNA-delivered, in silico predicted neoepitopes, to delay tumor growth and induce balanced T-cell responses in an additional tumor model and mouse strain.

Moved by the observation that neoepitope DNA immunization induces a modest anti-tumor effect in the B16F10 tumor model compared to CT26 discussed above, we explored potential underlying immunological resistance mechanisms by performing a multiparametric FACS-based immune characterization of B16F10 tumors. B16F10 tumors isolated from pB16F10–13-immunized mice presented immune phenotypic changes linked to improved outcomes, such as increased numbers of NK cells (Fig. 7i) and a marked reduction of intratumoral Myeloid-Derived Suppressor Cells (MDSCs) (Supplementary Fig. 4) as compared to mock DNA. In contrast, a significant enrichment of regulatory FoxP3+ CD4+ T cells (Fig. 7j) and limited presence of CD45+ immune cells and both CD4+ and CD8+ T cells.

Finally, to further investigate the limited anti-tumor protection conferred by neoepitope DNA immunization in the B16F10 model, we interrogated vaccine-induced, neoepitope-specific T cells for expression of exhaustion markers. MHC multimers loaded with a minimal epitope (SGFRYNVL) from the immunogenic B7 vaccine neoepitope were used to co-stain splenocytes for exhaustion markers PD-1, TIM-3, and LAG-3. B7 neoepitope-specific CD8+ T cells were readily detected in the spleens of mice vaccinated with pB16F10-13 but not mock DNA (Fig. 7f). Interestingly, the B7 multimer+ CD8+ T-cell fraction in the pB16F10–13 group showed a strong expression of TIM-3 combined with a modest upregulation of LAG-3, as compared to the multimer fractions of both pB16F10–13 and mock groups (Fig. 7g).

## Discussion

In the current era of personalized cancer immunotherapy, several approaches utilizing neoepitopes for active immunization are being pursued (*as reviewed extensively by Blass & Ott, 2021*^37^). The ability of neoepitope vaccines to induce or augment tumor-specific immune responses has been established in early clinical trials employing different vaccine formulations, hence personalized neoepitope vaccination is now positioned as a promising, yet costly and time-consuming investigational therapy in several cancer indications.

Preclinical models lack direct translatability to the clinic but nonetheless provide important mechanistic lessons about the dynamic interplay between evolving tumors and the immunological recognition that may lead to tumor regression. DNA-based vaccination has historically not provided much clinical success despite promising data from animal models^38^. However, multiple clinical trials involving personalized neoepitope DNA vaccination are currently ongoing^39^, most of which entail DNA delivery with electroporation or jet injectors and in combination with CPI therapy.

Our initial observation was that prophylactic immunization with DNA encoding a set of five in silico predicted neoepitopes provided protection against CT26 tumor challenge, whereas mock DNA did not. This was accompanied by convincing immunogenicity of neoepitope DNA vaccination which induced a balanced CD4+ and CD8+ T-cell response with preferential recognition of the mutated epitopes, not the wildtype sequences. In subsequent studies, we delineated via selective CD4+ or CD8+ T-cell depletion that CD8+ T-cells were fundamental to prevent tumor growth (potentially due to direct cytolytic activity of tumor-specific CD8+ T cells), while CD4+ T-cells played a role in shaping a potent CD8+ T-cell response. Mice depleted of CD4+ T cells had a diminished ability to inhibit CT26 tumor growth. We also observed a superior capacity to prevent tumor growth by delivering five neoepitopes contained in a single plasmid rather than either of the subgroup plasmids (neoepitopes 1-2 or 3–5). Consistent with observations from the T-cell depletion studies, the neoepitope-specific CD8+ T-cell immune response was hampered when the strongest CD4+ T-cell epitopes were not present in the immunization plasmid. Though the tumor cell lines used in this study do not express MHC class II, there are previous reports of the importance of CD4+ T cells and MHC class II-restricted neoepitopes in preclinical cancer immunotherapy^18,25,35^. The 27mer neoepitopes included in this study encompass multiple predicted MHC class I and II ligands with the potential to prime both CD4+ and CD8+ T cells.

Descriptions of CT26 neoepitope immunization in the literature report how in one case a single CD4+ T-cell inducing neoepitope could facilitate tumor control^25^, while another showed that potent tumor control from a group of five primarily MHC class II biased neoepitopes was, surprisingly, completely abrogated upon CD8+ T cell depletion^18^. Hence, there are mixed reports on the need and contributions of CD4+ and CD8+ T cells in the described syngeneic tumor models.

We did attempt an in vivo experiment with therapeutic administration of the neoepitope encoding DNA plasmid (i.e. after subcutaneous CT26 tumor inoculation) but were not able to show a significant anti-tumor effect. Improved methods of DNA vaccination can boost the immunogenicity and effect of these vaccines and we therefore expect that delivery via device such as electroporation or jet-injector, or otherwise improved DNA (e.g. APC targeting or co-delivery with cytokine-encoding plasmids) can enhance the effect against tumors in a therapeutic setting.

With our DNA platform, we incorporated 13 neoepitopes of 27 AA in length in the DNA plasmids, which facilitated complete protection from CT26 tumor establishment, and a broad and durable neoepitope-specific T-cell response. Interestingly, we demonstrated that these T-cell responses resulted from specific vaccination, as naïve mice or mock DNA-immunized mice did not respond to neoepitope presentation or stimulation. The potency and longevity of the platform were apparent as one dose of pCT26-13 could prevent CT26 tumor development in 38% of immunized mice when challenged with tumor cells 210 days after the priming immunization. The inclusion of multiple neoepitopes in cancer vaccine formulations is being pursued in both mice and humans^14,40–43^. The multiple neoepitope approach has the potential to induce several clones of tumor-recognizing T-cells, and broad recognition of tumor antigens is expected to have a greater protective potential in cancer. A plausible explanation for this is that the breadth of anti-tumor responses provides better protection if tumor variants that express a given mutation are eliminated because of immunoediting, or to account for the genetic heterogeneity of tumor cells. The presented data agree with previous studies establishing that far from all predicted neoepitopes are immunogenic^44^. Therefore, including multiple possible neoepitope targets improves the chances of raising a potential T-cell response. We furthermore assessed an in-house developed tool for DNA sequence optimization, and we found that the optimized sequences were functional and efficient in delivering multiple neoepitopes and conferring tumor control. In the context of personalized DNA vaccines, where each construct will have a patient-tailored neoepitope insert, suboptimal DNA designs can lead to long production times. The optimization tool that was utilized in the presented work is also used in the described ongoing phase 1/2 clinical trial, to attain fast and optimal DNA construct synthesis along with increased translation, to manufacture personalized vaccines for very ill patients in due time.

αPD-1 combination therapy with a suboptimal dose of neoepitope DNA vaccine showed a superior effect on tumor control and neoepitope immunogenicity relative to either monotherapy. This supports prior descriptions of the CT26 model, where αPD-1 monotherapy only had a modest ability to cause tumor regression. Neoepitope-specific T cells were observed at the highest frequency in blood, spleens, and tumors of pCT26-13 immunized mice, while αPD-1 monotherapy also elicited some, albeit of lower frequency, neoepitope-specific T-cells despite no neoepitope specific immunizations of these mice. This is suggestive of T-cell re-invigoration and epitope spreading events because of the αPD-1 mediated cytotoxic anti-tumor immune response. The presence of neoepitope-specific T cells that arise following αPD-1 monotherapy underlines the therapeutic relevance of our in silico predicted neoepitopes and support the notion that neoepitopes are important targets in CPI therapy of cancers.

We further applied the neoepitope DNA immunization in an additional model: B16F10 tumors syngeneic to C57BL/6 mice. Here, immunization with the in silico B16F10-predicted top 13 neoepitopes caused B16F10 tumor growth delay compared to mock DNA. To bridge our findings to published data from others we also applied a DNA plasmid encoding published control epitopes, however, this was inferior to the predicted top 13 neoepitopes both in terms of tumor delay and magnitude of specific T-cell responses. Interestingly, attempts in the literature to use DNA-formulated vaccines to deliver published B16F10 neoepitopes have resulted in CD8+ T-cell immunogenicity but a lack of tumor growth control^41^. In this model, both T-cell compartments have been scrutinized for their importance in other publications: some studies assign a key role to CD4+ T cells, as MHC class II-restricted neoepitope immunization results in B16F10 tumors elimination^18^, while a recent publication pointed to a single MHC class I-restricted epitope and CD8+ T cells able to facilitate B16F10 tumor control^45^.

Our investigation on immune resistance mechanisms that may underline the limited B16F10 tumor reduction driven by neoepitope vaccination demonstrated that the favorable immune changes of NK-cell enrichment and MDSC reduction in the tumor microenvironment (TME) occur in an immune-deserted environment, with limited T-cell infiltration and strong enrichment of regulatory FoxP3+ CD4+ T cells. The immunologically cold profile of B16F10 tumors has been described before^23^ and immunotherapy-driven rejection of established B16F10 tumors has been shown to require not only attenuation of immunosuppressive mechanisms but also enrichment of functional, tumor-specific T cells^18^. Thus, the limited reshaping of the TME after vaccination may explain the relatively weak tumor effect and underscores the requirement of a multifaceted TME immune modulation to attain enhanced tumor control. We also observed a significant enrichment of splenic, vaccine neoepitope-specific CD8+ T cells in vaccinated mice which, however, expressed modest and high levels of the exhaustion markers LAG-3 and TIM-3, respectively, as compared to bulk CD8+ T cells. It has been shown that the intratumoral, vaccine-induced, neoepitope-specific CD8+ T cells driving anti-tumor responses exhibit a less exhausted phenotype with lower expression of PD-1 and TIM-3^19^ and that the tumor size negatively correlates with the frequency of intratumoral PD-1+ TIM-3+ CD8+ T cells in another preclinical cancer model^46^. Although our data indicate upregulation in surface LAG-3 rather than PD-1 among neoepitope-specific T cells and the scrutinization of the splenic rather than the tumor compartment, the elevated levels of TIM-3 and LAG-3 among neoepitope-specific CD8+ T cells may reflect increased systemic immunosuppression with unfavorable implications for the capacity to control tumor growth.

Another unfavorable implication would be that immunoediting of the tumors may occur in vivo which can lead to the occurrence of antigen-loss tumor variants. Consequently, there could be a reduced surface presentation of T-cell antigens leading to evasion from immune recognition. This could be addressed by sequencing of in vivo grown tumors, comparing them to the in vitro expanded cell lines that were used for the neoepitope prediction.

Both the CT26 and B16F10 tumor models have been described to respond to checkpoint inhibitor monotherapy. It will be interesting to investigate the neoepitope DNA immunization in additional murine models of cancer, including cell lines that are less intrinsically immunogenic and do not respond well to checkpoint inhibition, such as 4T1 or Lewis lung carcinoma^23^.

Currently, nucleic acid-based vaccines are taking center stage in the development of prophylaxis and therapy for both infectious diseases and cancer. While several mRNA vaccines were approved for human use during the global efforts against the COVID-19 pandemic, there was also recently an authorization for emergency use of the first-ever DNA vaccine in humans: ZyCoV-D in India. A benefit of DNA vaccines is that they offer more amenable storage conditions than mRNA vaccines, which often entail a stringent cold chain, requiring logistics, monitoring, and ultra-cold freezers. A limitation of standard plasmid vectors is the high potential to be silenced in vivo or to be degraded by the intracellular machinery. New attempts to induce a more efficient immunological response to DNA vaccines with optimized transgene expression have shown encouraging results in preclinical studies and clinical trials and therefore hold great promise for human use. Approaches to reduce the extragenic spacer length by removal of the bacterial backbone components like in DNA minicircles or insertion of necessary bacterial components into introns (mini-intronic plasmids; MIPs) could circumvent the transgene silencing effects^47^. Another recent upgrade of the traditional plasmid vector with increased transgene expression is synthetic, linear, double-stranded DNA (doggybone, dbDNA, or oDNA). These emerging technologies may facilitate the further success of DNA vaccines for clinical use, along with benefit from physical devices or chemical delivery methods, e.g. electroporation, Jet-injectors, or gene guns (*reviewed in detail by Jorritsma* et al. *2016*^48^).

The data presented here support the concept that DNA offers a potent and versatile neoepitope delivery approach allowing the encoding of multiple neoepitopes in a single formulation.

Importantly, a balanced T-cell response was observed to be correlated with efficient anti-tumor immunity after DNA vaccination, which is why we argue it is favorable for in silico selected neoepitopes to contain both MHC class I and -II ligands, even for tumors that are MHC-II negative.

## Methods

### Mice

6- to 8-week-old BALB/c JrJ and C57BL/6 JrJ female mice were acquired from Janvier Labs (France). The mice were acclimated for one week before the initiation of the experiments. The experiments were performed in accordance with relevant guidelines and regulations, conducted under license 2017-15-0201-01209 from the Danish Animal Experimentation Inspectorate in accordance with the Danish Animal Experimentation Act (BEK nr. 12 of 7/01/2016), which is compliant with the European directive (2010/63/EU).

### Cell lines

BALB/c syngeneic colon cancer cell line CT26 (#CRL-2638) and C57BL/6 syngeneic B16F10 melanoma cell line (#CRL-6475) were purchased from ATCC and cultured as per supplier’s instructions at 37 °C and 5% CO_2_. All cell lines tested negative for mycoplasma infection.

### CT26 neoepitope prediction and selection

CT26 somatic mutations and gene expression values were retrieved from the previous work^34^. Somatic mutations were filtered to only include non-synonymous mutations. Reference source protein sequences were downloaded using the Biopython Entrez module. For each somatic mutation, a 29mer neoepitope sequence was extracted from the corresponding source protein, with the mutated amino acid (AA) at position 15. If the mutated AA was located within 15 AA of the ends of the source protein, a shorter neoepitope sequence was extracted instead. If the extracted neoepitope sequence was shorter than 15 AAs, it was discarded. Furthermore, if the annotated wildtype AA was not found at the mutated position in the reference source protein, the neoepitope was also discarded.

For each neoepitope sequence, NetMHCIIpan^49^ was used to generate MHC class II binding predictions for all overlapping 15mers towards the mouse MHC class II molecule H-2-IA^d^ (BALB/c). The 15mer with the best NetMHCIIpan %rank score was selected as representative of the neoepitope. Neoepitopes with a gene expression >20 RPKM (Reads Per Kilobase of the transcript, per Million mapped reads) were ranked based on their best NetMHCIIpan %rank score and the top 5 and top 13 were selected for in vivo evaluation. Finally, the N- and C-terminal AAs of the selected neoepitopes were truncated to convert the 29mers to the final 27mer neoepitope sequences.

### Next-generation sequencing and analysis

DNA and RNA from B16F10 cells and DNA from a C57BL/6 tail tissue sample were extracted using DNAeasy Blood and Tissue Kit (Qiagen, #69504) and RNAeasy Mini Kit (Qiagen, #74104), respectively, and sent to GenomeScan (Leiden, The Netherlands) for whole exome sequencing (WES) and mRNA sequencing. Exome capture was performed using the Agilent SureSelectXT Mouse All Exome capture kit. RNA-seq libraries were prepared using the NEBNext Ultra Directional RNA Library Prep Kit for Illumina. All libraries were sequenced on an Illumina HiSeq 4000. 60 M clusters (2 × 150 bp) were sequenced for each exome library and 50M clusters were generated for the RNA-seq library. Raw fastq files were preprocessed with Cutadapt^50^. WES reads were mapped to the GRCm38 mouse reference genome from Ensembl^51^ using BWA-MEM^52^ and PCR duplicates were removed using samtools^53^. Germline variants were identified using DeepVariant^54^. A bulk list of somatic variants was identified using LoFreq, Mutect2, Strelka, and SNVSniffer. This list of somatic variants was then filtered using a proprietary somatic variant caller developed at Evaxion Biotech, generating a Somatic probability score for each variant.

Tumor purity was estimated using Sequenza^55^ and used to correct the variant allele frequency (VAF) of each somatic variant. Corrected VAFs, combined with the Somatic probability score, were used to calculate a Clonal probability score for each somatic variant. RNA reads were mapped using STAR^56^ and transcript isoform expression was quantified using RSEM^57^. Transcript isoform expression values were combined with the RNA VAF of each somatic variant to calculate variant transcript isoform expression values.

### B16F10 neoepitope prediction and selection

The variant effects of both somatic- and germline variants were annotated using VEP^58^. Somatic- and germline variants annotated to alter AA sequences were introduced into the corresponding reference protein sequences resulting in a tumor-specific protein sequence. Neoepitope sequences of 27 AAs are extracted around each AA change caused by a somatic variant. Proteins affected by germline frameshifts or splice-site-affecting variants were disregarded. A neural network-based MHC ligand prediction tool developed by Evaxion Biotech was used to predict MHC ligands in identified neoepitopes. The tool generates oligomers from each peptide and generates an MHC presentation prediction for each designated MHC allele. The best prediction was used to represent the likelihood of MHC ligand presentation. This procedure was done separately for MHC class I (MHC-I) and MHC class II (MHC-II). MHC-I predictions were generated for oligomers of size 8, 9, 10, and 11 and MHC-II predictions were generated for oligomers of size 15. Raw MHC predictions were integrated with variant transcript isoform expression values to generate calibrated MHC ligand probability scores.

An immunogenicity probability score (*I*) was generated for each neoepitope based on a property distance score between the neoepitope and wild-type peptide. The property distance score was calculated as the summed Euclidian distance of each AA pair in the neoepitope and wildtype peptide, where each AA is defined by a 5-dimensional vector representation of its physical–chemical properties.

Neoepitopes were ranked based on the product of the Somatic, Clonality, MHC ligand, Immunogenicity probability scores calculated as described above, generating a prioritized list of neoepitopes. 13 neoepitopes were selected starting from the top of the list, but subject to the following exclusion criteria: (1) the neoepitope was not expressed, (2) the neoepitope did not contain either a predicted MHC-I ligand or a predicted MHC-II ligand, (3) the neoepitope arose from the same mutation as a previous neoepitope, (4) the best predicted MHC-I and MHC-II ligands matched the best-predicted ligands from a previously selected neoepitope.

### Neoepitope DNA insert optimization tool

A bioinformatics tool was developed at Evaxion Biotech and employed to optimize the neoepitope insert for several DNA plasmids used for in vivo immunization for optimal insert synthesis and expression. The *Evaxdesign* tool optimizes the sequence’s codon adaptation index (CAI) and GC content, eliminates the risk of repeats, misplaced TATA boxes, and premature poly-A tails, and finally performs optimization of secondary RNA structure. In brief, a cohort of DNA constructs is generated by reverse translation of neoepitope protein sequence through sampling from an organism-specific codon table weighted by codon usage. These sequences are then randomly concatenated with a predefined set of linker sequences all encoding the same GS linker. This set of predefined linkers was created ensuring maximal DNA sequence diversity to minimize the risk of DNA repeats. Then, the necessary sequence features are added, such as the Kozak sequence and restriction sites to each construct. The tool generates 10,000 DNA constructs and evaluates each construct’s CAI, GC content, and RNA minimum free energy. Then follows assigning of dynamic thresholds for CAI and GC content, which eliminates 99% of the constructs. We then evaluate the RNA minimum free energy for the remaining top 1% and select the construct with the highest energy. Once the final construct has been selected the optimized sequence can be manufactured.

### Synthetic peptides

For the study of the non-synonymous mutations encoded by the DNA constructs and the corresponding wild types, the corresponding 27mer peptides featuring the mutated AA in the center were synthesized by Pepscan (Lelystad, Netherlands) or Genscript (New Jersey, USA). The lyophilized peptides were dissolved in dimethyl sulfoxide (DMSO) (Merck, #D8418) to a concentration of 10 mg/mL and used in ex vivo assays. The sequences of the different epitopes are given in Supplementary Tables 1 and 2.

### DNA plasmids

#### Research grade

Codon-optimized DNA inserts encoding CT26 or B16F10 neoepitope sequences in tandem connected by glycine and serine linker elements and containing a Kozak consensus sequence to effectively initiate translation were cloned in research-grade standard pVAX1 (Thermo Fischer, #V26020) or pTVG4 DNA plasmid. The plasmids contain a CMV-driven expression vector and the kanamycin resistance gene for selection. The pTVG4 DNA vector was generated from the standard plasmid pUMVC3 acquired from Aldevron (North Dakota, USA, #4010) by cloning two copies of a 36-bp CpG-rich immunostimμLatory DNA sequence (ISS) containing the 5’-GTCGTT-3’ motif downstream of the multi-cloning site^59^. pTVG4 DNA plasmid was upscaled by Aldevron. Empty pVAX1 and pTVG4 DNA vectors with no cloned neoepitope DNA insert were used as controls.

#### Clinical grade

Empty or CT26 neoepitope-encoding pTVG4 DNA plasmids designed as described above were upscaled at COBRA Biologics as clinical-grade DNA.

### In vivo immunization

50 or 100 μg of research-grade DNA were formulated with the block co-polymer poloxamer 188 (gifted by BASF, Germany) to a final vaccine concentration of 3% in an isotonic buffer immediately prior to injection. 3, 25, or 50 μg of clinical-grade DNA were formulated with GMP-formulated poloxamer 188 (gifted by BASF, formulated at HALIX) to a final vaccine concentration of 3% in PBS. Mice were immunized weekly in the left and right tibialis anterior muscles (i.m.) with 2 × 50 μl of DNA (between 1 and 9 total immunizations). Mice were prophylactically immunized relative to tumor cell inoculation. To deplete T-cell subsets in vivo, groups of mice were injected every 6–7 days in the peritoneal cavity with 200 μL PBS with 200 μg anti-mouse CD8 (BioXcell, clone 02.43, #732020F1), anti-mouse CD4 (BioXcell, clone GK1.5, 728319D1) or isotype control antibody (BioXcell, clone LTF-2, # 707119D1B) starting before the DNA immunizations. For the CPI therapy combination studies, groups of mice were injected intraperitoneally with 100 μL PBS with 200 μg anti-mouse PD-1 (BioXcell, clone RMPI-14, #800121A1ZB) or with isotype control antibody (BioXcell, clone 2A3, #BP0089). The anti-PD-1 antibody administration was initiated upon detection of palpable tumors and was replenished every 3–4 days.

### Tumor challenge experiments

At the day of tumor cell inoculation (defined as study day 0), in vitro expanded CT26 or B16F10 cells were trypsinized and washed twice in a serum-free medium. CT26: 5 × 10^5^ or 2.5 × 10^5^ cells per 100 μL RPMI, for B16F10: 1.5 × 10^5^ cells per 100 μL DMEM. Tumor cells were inoculated subcutaneously in the right flank of mice. Once established, the tumor diameters were measured three times per week with a digital caliper. The tumor volumes were calculated using the following formula: ${\rm{tumor}}\,{\rm{volume}}=\frac{\pi }{6}\ast {({{d}}_{1}\ast {{d}}_{2})}^{3/2}$, where *d*_1_ and *d*_2_ are orthogonal diameters of the tumor.

The mice were euthanized through cervical dislocation when the majority of tumors in the control groups reached the maximum allowed size of 12 mm diameter in either direction or upon reaching humane endpoints.

Upon euthanization, spleens from mice with tumors representative of their group’s average tumor size were collected in cold RPMI supplemented with 10% FCS (from here on: R10), followed by processing to single cell suspensions via GentleMACS processing (Miltenyi Biotec, C-tubes #130-096-334 and Dissociater #130-093-235) and passage through a 70 μm filter (Corning, CLS431751).

Splenocytes were cryopreserved in FCS supplemented with 10% DMSO (Merck, #D8418).

### Peptide re-stimulation and IFNγ Enzyme-linked ImmunoSpot (ELISpot)

PVDF membrane plates (Merck Millipore, #MAIP4510) first activated with 35% v/v ethanol were coated overnight with 5 μg/mL anti-IFNγ capture antibody (BD, #51-2525KZ, 1:200). 5 × 10^5^ splenocytes were plated per well and stimulated with 5 μg/mL synthetic peptides or unstimulated in a total volume of 200 μL R10 medium. Cells were incubated overnight at 37 °C and 5% CO_2_. To detect IFN-γ secreting cell spots, anti-IFNγ detection antibody (BD, #51-1818KA, 1:250), streptavidin-HRP enzyme (BD, #557630), and AEC chromogen substrate (BD, #551951) were applied sequentially following the manufacturer’s protocol. ELISpot plates were imaged and IFNγ spots were counted using an ELISpot reader (Cellular Technology, Ltd).

### Peptide re-stimulation and intracellular cytokine staining (ICS)

2 × 10^6^ splenocytes plated in round-bottomed, 96-well culture plates (Corning, #3799) were stimulated with 5 mg/mL synthetic peptides or left unstimulated in a total volume of 200 μL R10 medium. Two hours after initiation of stimulation, protein transport was inhibited by the addition of brefeldin A (GolgiPlug, BD #555029) and monensin (GolgiStop, BD #554724), and cells were incubated overnight at 37 °C and 5% CO_2_. Cells were washed, incubated with Fc-receptor (FcR) blocking anti-CD16/32 (Biolegend, #101301, 1:50) for 10 min at 4 °C and then surface stained for CD3e (FITC, Biolegend #100305, 1:800), CD4 (PE-Cy7, BD #552775, 1:600), CD8 (BV786, BD #563332, 1:250), and viability dye (GloCell™ Fixable Viability Dye, Stem Cell Tech #75010, 1:1000) for 30 min at 4 °C. Hereafter cells underwent fixation and permeabilization (eBioscience, #00-8222-49 and #00-8333-56) before being stained intracellularly for IFNγ (BV650, BD #563854, 1:130) and TNFα (BV421, BD # 566287, 1:130) for 30 min at 4 °C.

Flow cytometry was performed on FACS Celesta (BD) and the frequencies of cytokine-producing CD4+ and CD8+ T-cells were determined in FlowJo software (version 10.8.0), see Supplementary Fig. 5 for gating strategy.

### MHC multimer staining for detection of neoepitope-specific CD8+ T cells

All MHC multimers were purchased from Tetramer Shop (Denmark). MHC multimers specific to the CT26 tumor model were refolded with peptides at Tetramer Shop, while MHC multimers specific to the B16F10 tumor model consisted of empty and peptide-receptive H-2K^b^ molecules, tetramerized via PE-conjugated streptavidin (Tetramer Shop, #MKb-016), loaded with peptides at Evaxion Biotech (minimal epitope SGFRYNVL derived from the vaccine-contained B7 27mer sequence ELCRVCGDKASGFRYNVLSCEGCKGFF, or negative control peptide SIINFEKL derived from Ovalbumin 257–264). To monitor the development of vaccine neoepitope recognition in BALB/c mice with the CT26 tumor model MHC multimers comprising H-2K^d^ molecule loaded with the restricted minimal epitope KFKASRASI derived from the vaccine-contained C1 27mer sequence (QIETQQRKFKASRASILSEMKMLKEKR), or comprising H-2D^d^ molecule loaded with the restricted minimal epitope SQPSYATYL derived from the vaccine-contained C2 27mer sequence (VILPQAPSGPSYATYLQPAQAQMLTPP) were used to stain tail vein blood for the presence of neoepitope-recognizing CD8+ T-cells. Briefly, 50 μL tail vein blood was collected in EDTA-coated tubes (Sarstedt, #20.1278.100) and then transferred to deep, 96-well plates (Sigma, #575653). Cells were incubated with FcR blocking anti-CD16/32 (Biolegend, #101301, 1:50) for 10 min at 4 °C, then stained with 5 μl of MHC multimer (PE- or BV421-conjugated, Tetramer Shop custom products), and centrifuged at 3300×*g* for 5 min. Cells were incubated for 15 min at 37 °C. Cells were then surface stained for CD3e (FITC, Biolegend #100305, 1:800), CD4 (PE-Cy7, BD #552775, 1:600), and CD8 (BV786, BD #563332, 1:250) for 30 min at 4 °C. Finally, cells underwent one-step fixation/red blood cell lysis (eBioscience #00-5333-57) before acquisition on FACS Celesta and determination of MHC multimer+ CD8+ T-cells, see Supplementary Fig. 6 for gating strategy.

When splenocytes were used as input material for MHC multimer staining, 2 × 10^6^ splenocytes were thawed and plated in round-bottomed, 96-well culture plates (Corning, #3799) followed by the addition of FcR blocking anti-CD16/32 (Biolegend, #101301) and then stained with 5 μl of PE-conjugated MHC multimers (Tetramer Shop custom products for CT26 minimal epitopes as described above or loaded with B16F10 minimal epitopes in-house as per vendor’s instructions), as described in the prior paragraph. Cells were then surface stained for CD3e (FITC, Biolegend #100305, 1:800), CD4 (PE-Cy7, BD #552775, 1:600), CD8 (BV786, BD #563332, 1:250) and viability dye (GloCell™ Fixable Viability Dye, Stem Cell Tech #75010, 1:1000) for 30 min at 4 °C. When MHC multimer staining was combined with exhaustion marker staining, antibodies for PD-1 (BV605, BD #748267, 1:400), TIM-3 (PE-CF594, BD #566998, 1:100), and LAG-3 (BV711, BD #563179, 1:100) were also included in the above staining. Flow cytometry was performed on FACS Celesta (BD) and the frequencies of the immune populations were determined in FlowJo software (version 10.8.0), see Supplementary Fig. 7 for the gating strategy.

### Generation of tumor digests for ex vivo assays

Isolated tumors from 5 to 7 animals from selected groups were dissociated into single-cell suspensions with a cocktail of tumor dissociation enzymes (Miltenyi Biotech #130-096-730) and filtered through 70 μm cell strainers according to the instructions of the manufacturer. Tumor digests single cell suspensions were subjected to MHC multimer staining, as described in the paragraph above, see Supplementary Fig. 8 for the gating strategy.

Regarding the immune phenotyping experiments, B16F0 tumor digests were incubated with FcR blocking anti-CD16/32 (Biolegend, #101301, 1:50) for 10 min at 4 °C followed by surface staining for CD45.2 (PerCP-Cy5,5, BD #552848, 1:400), CD3e (FITC, Biolegend #100305, 1:800), CD4 (PE-Cy7, BD #552775, 1:600), CD8 (BV786, BD #563332, 1:250), viability dye (GloCell™ Fixable Viability Dye, Stem Cell Tech #75010, 1:1000), NK1.1 (BV605, Biolegend #108753, 1:200), CD11b (BV711, BD #563402, 1:800), Ly6G (BV421, Biolegend #127627, 1:800) for 30 min at 4 °C. Cells then underwent fixation and permeabilization through addition of Foxp3 Fixation/Permeabilization solution prepared by mixing one part of the concentrate with three parts of the diluent (Foxp3/Transcription Factor Staining Buffer Set, eBioscience™ #00-5523-00) for 60 min at 4 °C. Cells were then stained intranuclearly for FoxP3 (PE-eFluor610, Thermo Fischer, #61-5773-82, 1:400) in permeabilization buffer (eBioscience™ #00-8333-56) for 30 min at 4 °C. Flow cytometry was performed on FACS Celesta (BD) and the frequencies of the immune populations were determined in FlowJo software (version 10.8.0), see Supplementary Fig. 9 for gating strategy.

### Statistical analyses

GraphPad Prism 9 for Mac OS X was used for graphing, statistical analyses, and tools. Data were subjected to Kolmogorov–Smirnov test for normality (alpha = 0.05). Parametric data were analyzed by ordinary ANOVA with Sidak’s multiple comparison correction. Non-parametric data were analyzed by Mann–Whitney test (if two comparisons) or Kruskal–Wallis test with Dunn’s multiple comparison correction (if more than two comparisons). For all results, the following levels of statistical significance are applied: *p < 0.05, **p < 0.01, ***p < 0.001, ****p < 0.0001. All statistical tests were two-sided. Measurements were taken from distinct samples but for tumor growth monitoring the same samples were measured repeatedly over time. Data variation (error bars) represent the standard deviation (SD), except for tumor growth curves and area under the curve plots, where the variation is instead depicted as the standard error of the mean (SEM).

### Reporting summary

Further information on research design is available in the Nature Research Reporting Summary linked to this article.

## Supplementary information

Supplemental Information

REPORTING SUMMARY

## Data Availability

The datasets generated during and/or analyzed during the current study are available from the corresponding author upon request. B16F10 NGS data is available via NCBI Sequence Read Archive (SRA) under accession PRJNA934871.

## Code availability

The code and algorithms used during the current study are available from the corresponding author upon reasonable request.
