# Supplementary material for: DNA based neoepitope vaccination induces tumor control in syngeneic mouse models
Source: NPJ Vaccines. 2023 May 27;8:77. doi: 10.1038/s41541-023-00671-5 (PMC10224666; doi:10.1038/s41541-023-00671-5)
Supplement: Supplementary file 1 — Supplemental Information [file 41541_2023_671_MOESM1_ESM.pdf]

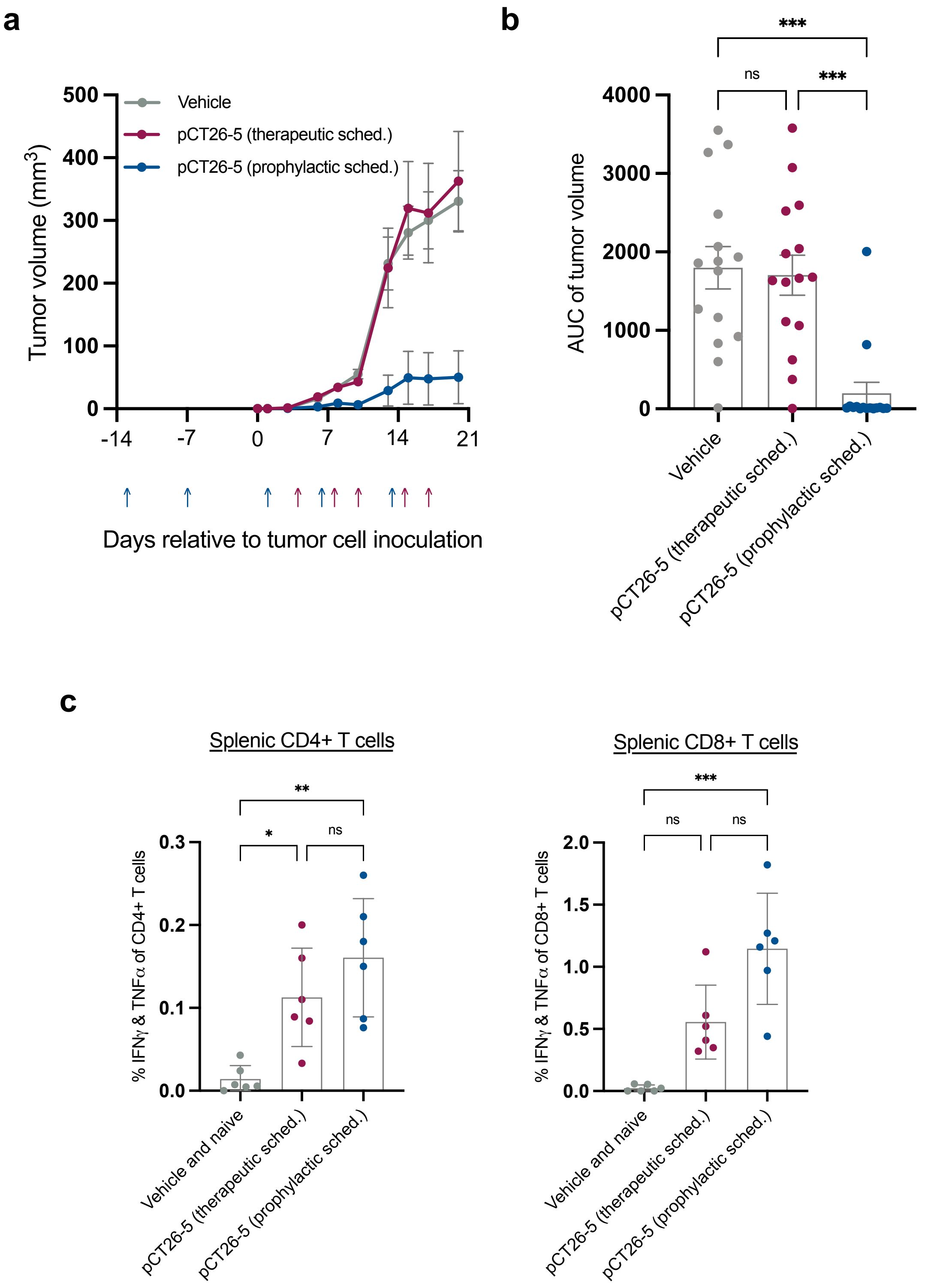

Supplementary Figure 1. pCT26-5 vaccinated mice can control tumor via prophylactic but not therapeutic immunization schedule. (a) Groups of n = 15 BALB/c mice were immunized i.m. either prophylactically (blue arrows indicate immunization days, starting from day -13) or therapeutically (dark red arrows indicate immunization days, starting from day 4) with 100 μg of pCT26-5 DNA plasmid or poloxamer ('vehicle') and s.c. inoculated on day 0 with 5 x 10^5 CT26 tumor cells. Vehicle was injected same days as prophylactic schedule. A group of age-matched n = 5 BALB/c mice received no vaccination or tumor cell inoculation ('naive'). **(b)** Tumor volume AUC for individual mice by group (mean ± SEM). **(c)** pCT26-5 peptide pool re-stimulation and ICS on single cell suspensions of splenocytes (n = 3-6 mice per group), showing cytokine double positive CD4+ (left) and CD8+ (right) T cells (mean ± SD). *Statistics*: Kruskal-Wallis test with Dunn's multiple comparisons correction (b and c) *$p < 0.05$, **$p < 0.01$, ***$p < 0.001$. Abbreviations; schedule: sched.

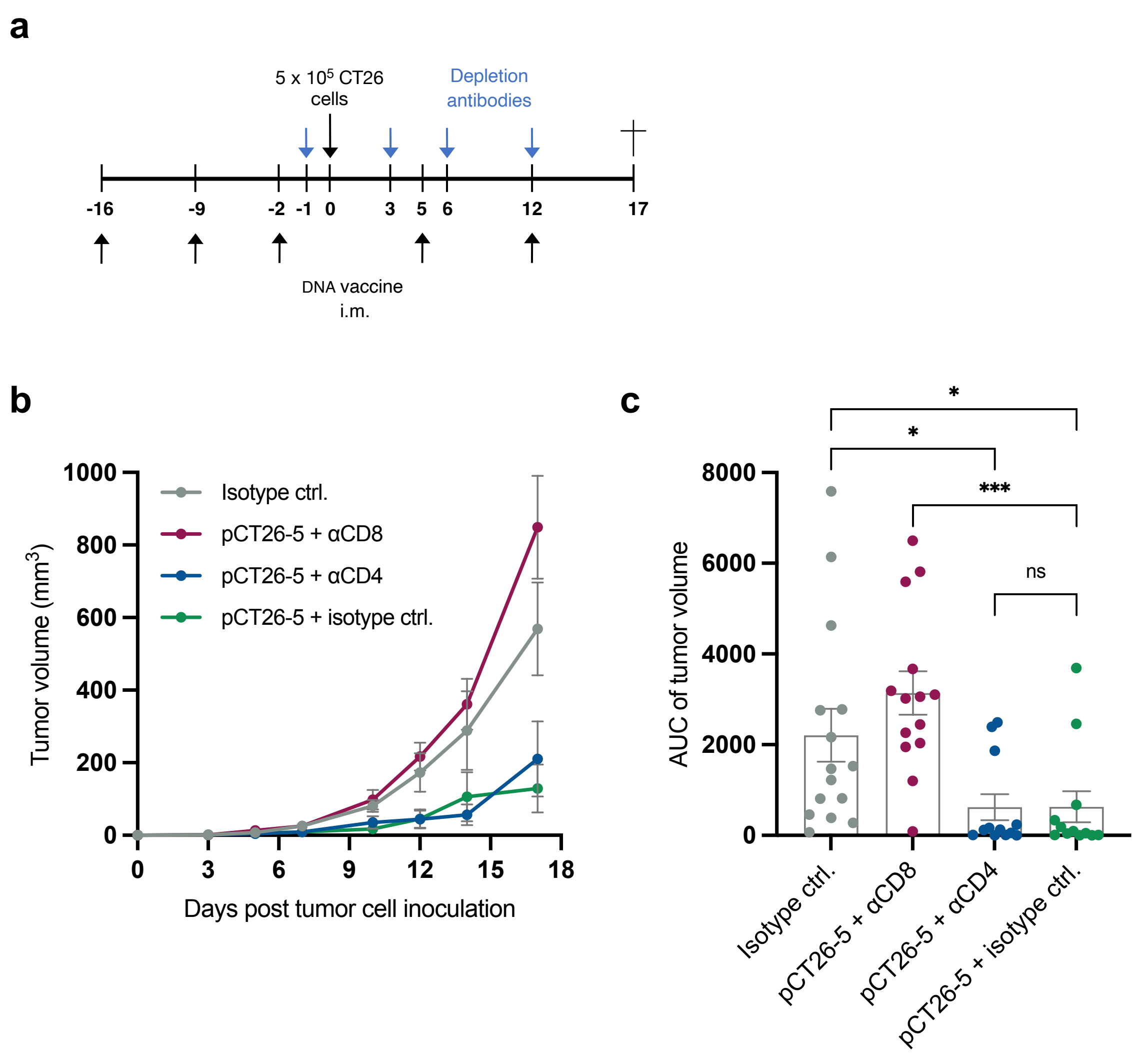

Supplementary Figure 2. CD8+ T cells are indispensable for anti-tumor capacity after neoepitope DNA immunization. (a) Representation of the timeline in the *in vivo* experiment with "late" T-cell depletion. Groups of n = 12-15 BALB/c mice were immunized prophylactically with 50 μg of pCT26-5 DNA before s.c. inoculation with CT26 tumor cells. One day before tumor inoculation, mice were administered antibodies to selectively deplete CD4+ or CD8+ T cells (or isotype control antibody) via i.p. administration. **(b)** Group mean tumor growth curves (in mm^3) ± SEM. **(c)** Tumor volume AUC for individual mice by group (mean ± SEM). *Statistics*: Kruskal-Wallis test with Dunn's multiple comparisons correction (b) *$p < 0.05$, **$p < 0.01$, ***$p < 0.001$.

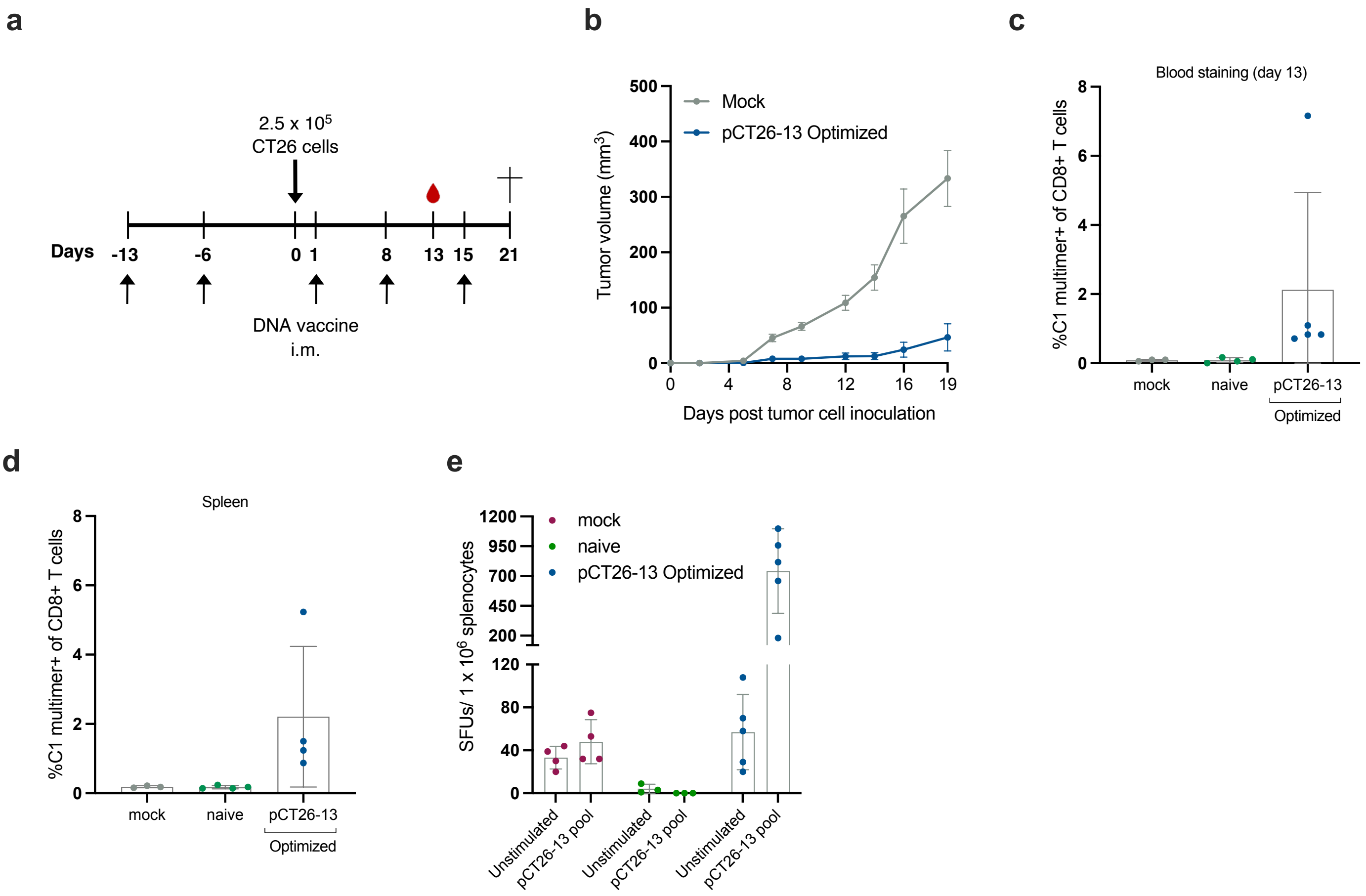

Supplementary Figure 3. **pCT26-13 induces *de novo* neoepitope-specific T-cell responses in vaccinated mice. (a)** Representation of the timeline in the *in vivo* experiment. Groups of n = 13 BALB/c mice were immunized prophylactically with 50 μg of either sequence optimized pCT26-13 DNA plasmid or mock DNA before s.c. inoculation with CT26 tumor cells. A group of age-matched n = 4 BALB/c mice received no vaccination or tumor cell inoculation ('naive'). To measure the frequency of neoepitope specific CD8+ T cells, **(c)** tail vein blood collected in EDTA coated tubes on study day 13 and **(d)** single cell suspensions of splenocytes generated upon sacrifice of the mice in the end of the study were stained with neoepitope C1-MHC multimer to detect specific CD8+ T cells (n = 3-4 mice per group). **(e)** pCT26-13 peptide pool re-stimulation and IFNγ ELISpot on single cell suspensions of splenocytes (n = 3-5 mice per group).

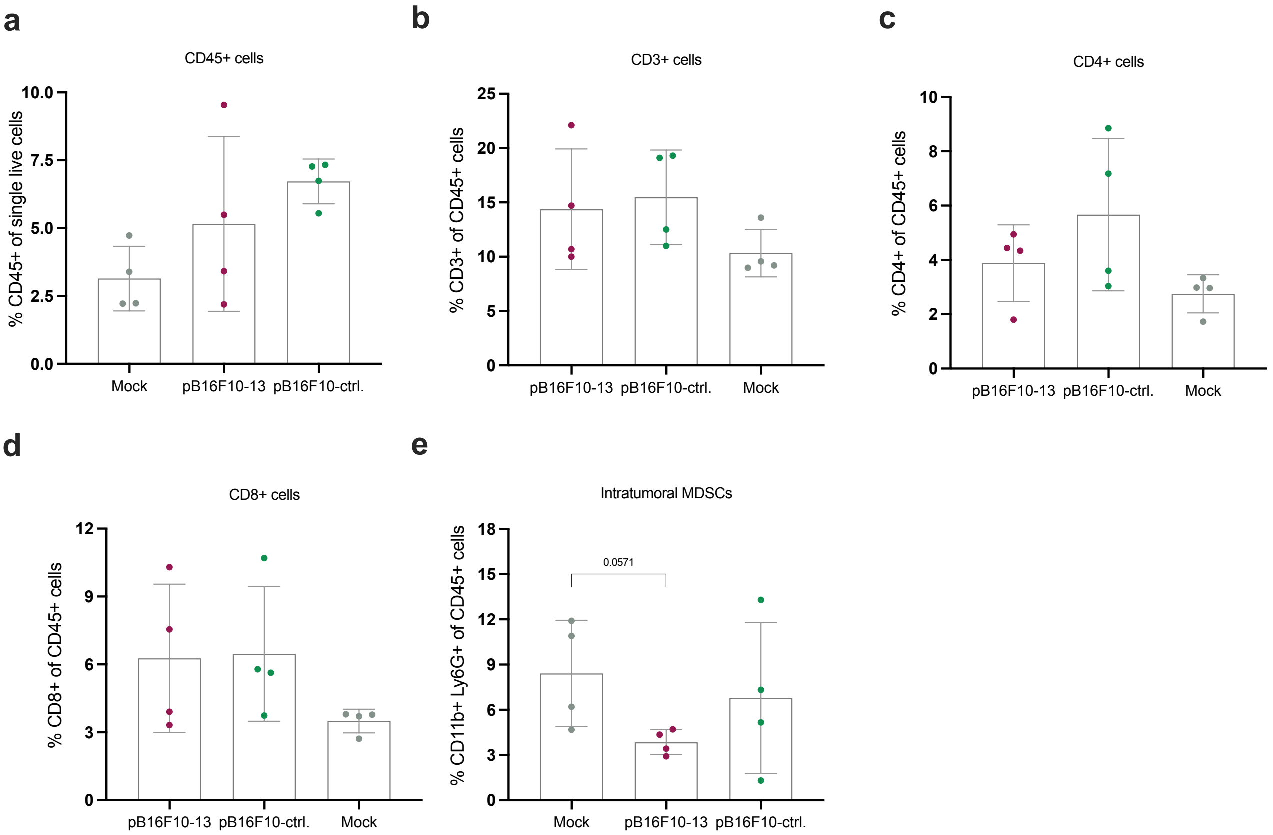

Supplementary Figure 4. B61F10 tumor digests from mice immunized with pB16F10-13 bear an immunological cold phenotype. Single cell suspensions of B16F10 tumor digests prepared from mice immunized with pB16F10-13, pB16F10-ctrl. or mock DNA (n = 4 per group) were stained with dead/live cell viability dye and antibodies specific to murine CD45, CD3, CD4, CD8 and CD11b/Ly6G markers to allow for quantification of intratumoral **(a)** CD45+, **(b)** CD3+, **(c)** CD4+, **(d)** CD8+ and **(e)** MDSCs, respectively. *Statistics*: Kruskal-Wallis test with Dunn's multiple comparisons correction (e) $^*p < 0.05$, $^{**}p < 0.01$, $^{***}p < 0.001$.

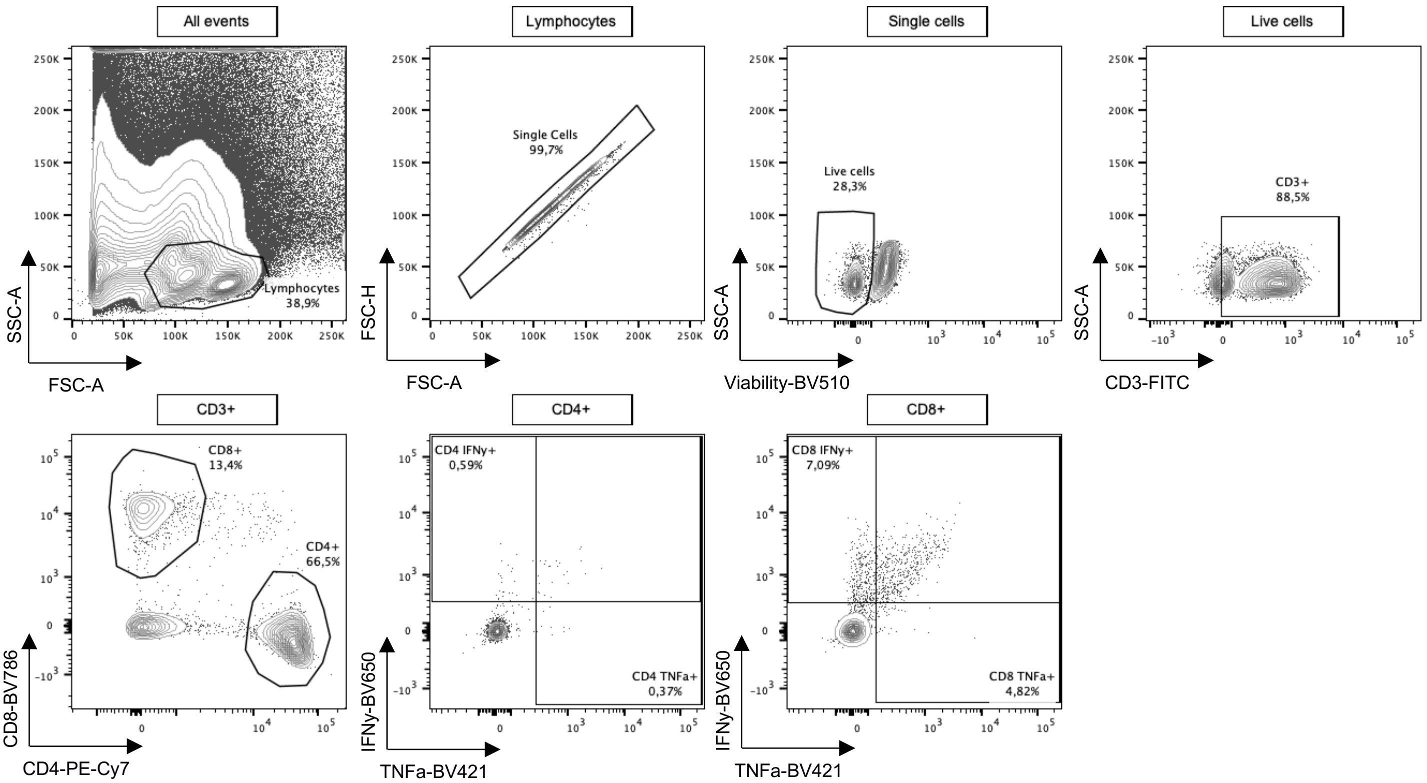

Supplementary Figure 5. FACS gating strategy. FACS gating strategy used for samples after peptide re-stimulation and intracellular cytokine staining of splenocytes.

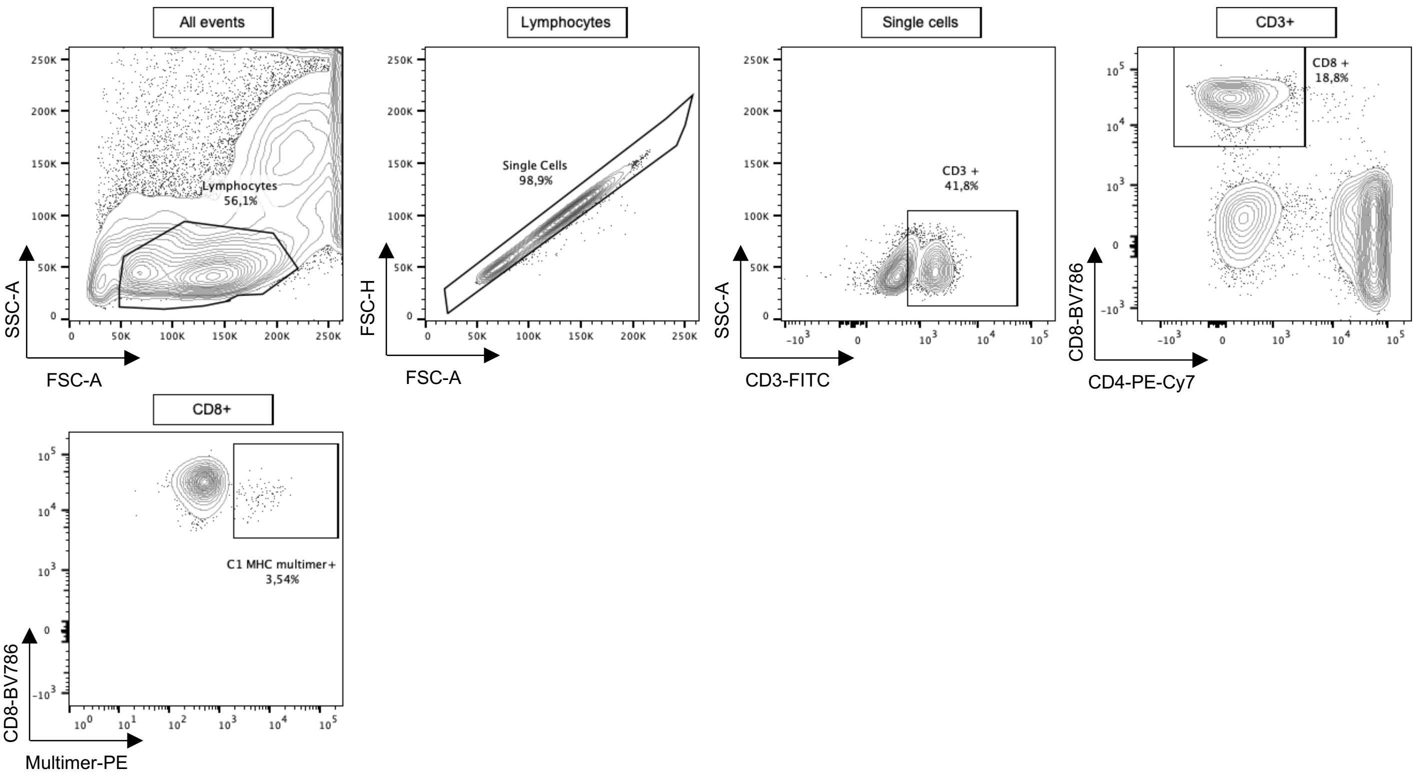

Supplementary Figure 6. FACS gating strategy. FACS gating strategy used for samples after MHC multimer staining of tail vein blood. Multimer-positive gate was set based on fluorescence minus one (FMO) controls and staining of blood from naïve control mice.

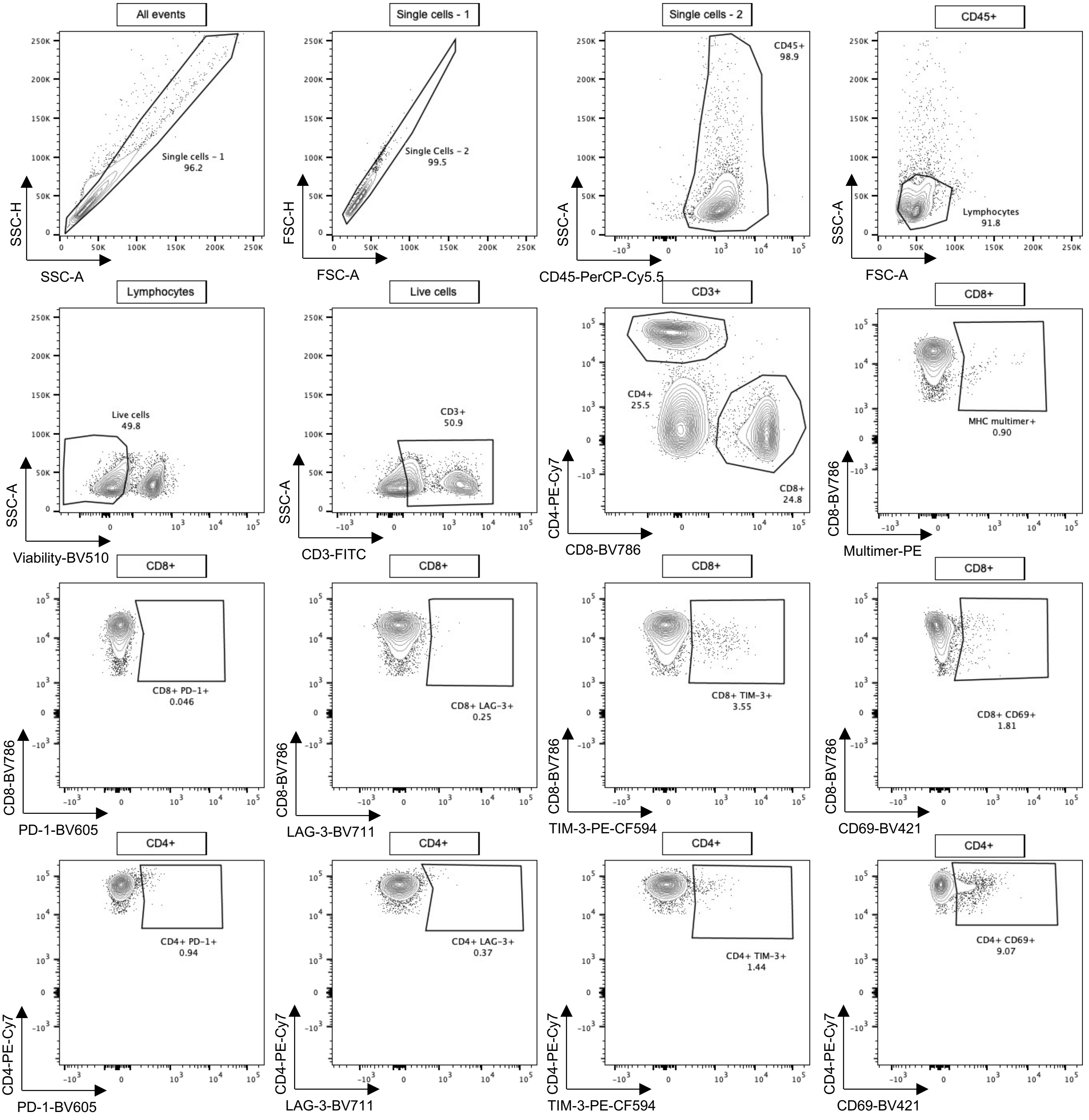

Supplementary Figure 7. FACS gating strategy. FACS gating strategy used for samples after MHC multimer and exhaustion marker surface staining of splenic single cell suspensions.

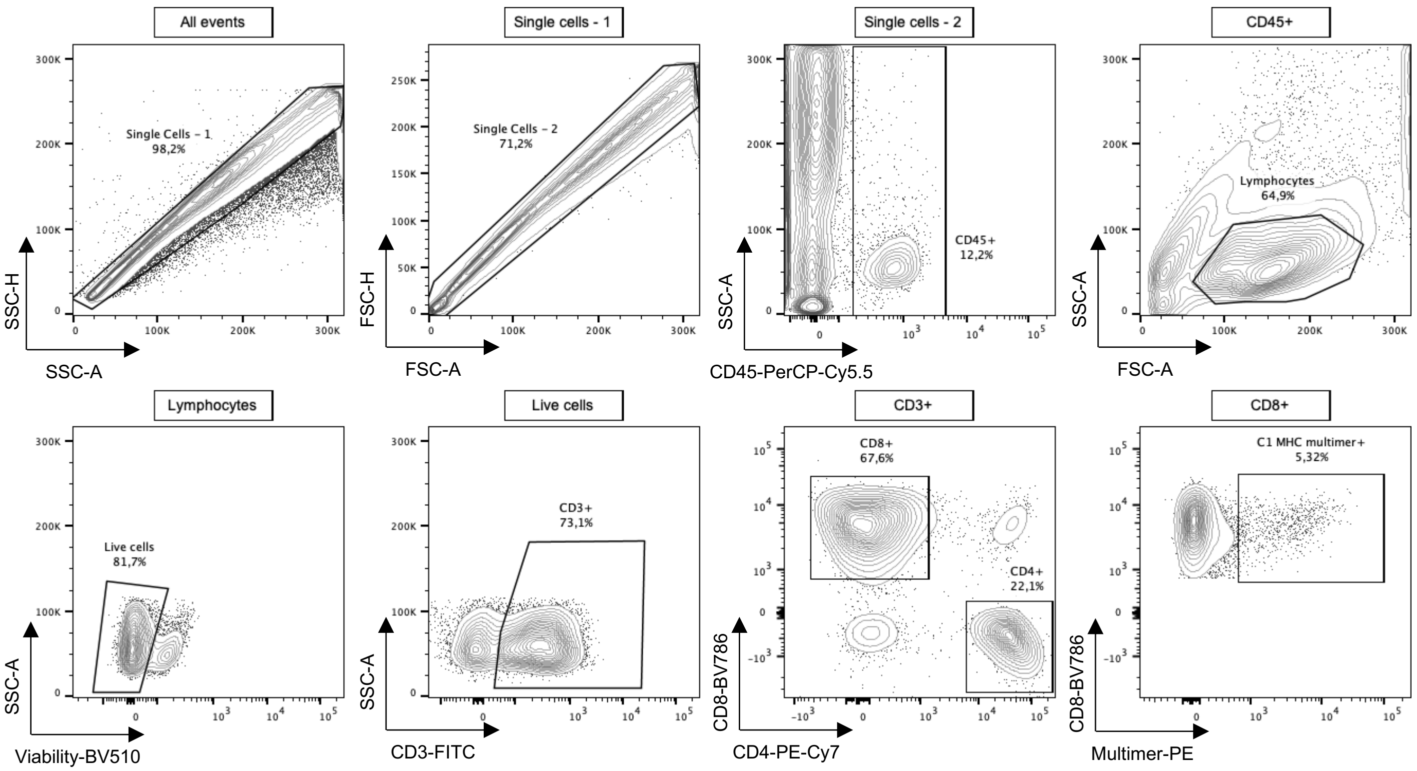

Supplementary Figure 8. FACS gating strategy. FACS gating strategy used for samples after MHC multimer staining of CT26 tumor single cell suspensions.

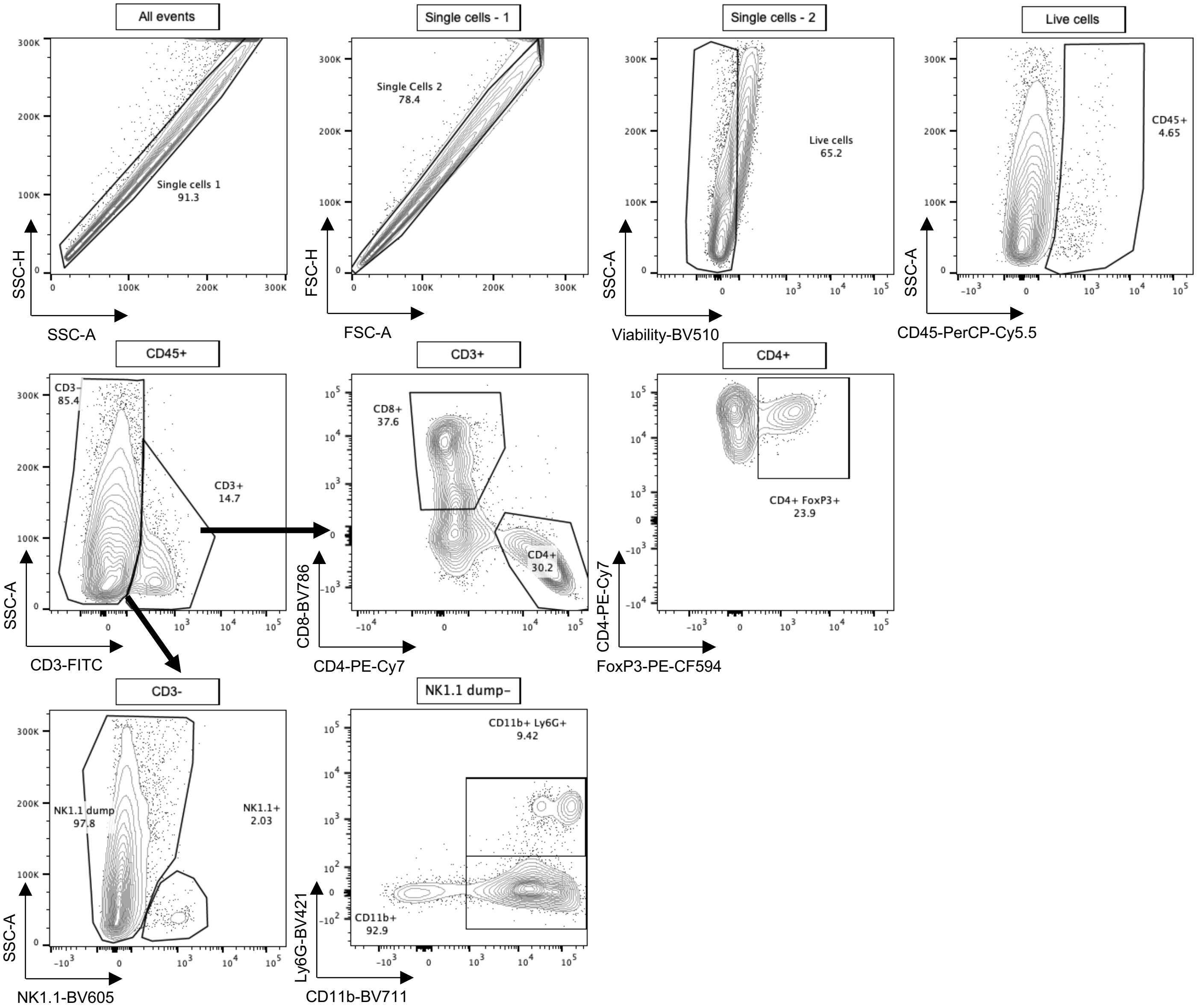

Supplementary Figure 9. FACS gating strategy. FACS gating strategy used on immune phenotyping stainings of singe cell suspensions of B16F10 tumor digests.

Supplementary Table 1. Overview of CT26 neoepitopes used for *in vivo* studies and *ex vivo* analyses. Bold amino acid denotes the position of the point mutation. Blue amino acids show the best predicted MHC-I neoepitope. Underlined amino acids show the the best predicted MHC-II neoepitope.

ID	Gene	AA sequence used for immunization	Substitution (WT, AA#, Mut)	DNA plasmids
C1	*Smc3*	QIETQQRKFKASR**A**SILSEMKMLKEKR	D733A	pCT26-5 & pCT26-13
C2	*E2f8*	VILPQAPSGPSYA**T**YLQPAQAQMLTPP	I522T	
C3	*Ppp6r1*	RLHVVKLLASALS**T**NAAALTQELLVLD	A368T	
C4	*Ubqln1*	DTLSAMSNPRAMQ**V**LLQIQQGLQTLAT	A456V	
C5	*Ppp6r1*	DGQLELLAQGALD**N**ALSSMGALHALRP	D309N	
C6	*Lin7c*	GEVPPQKLQALQR**A**LQSEFCNAVREVY	V41A	
C7	*Uchl3*	KKFMERDPDELRFN**T**IALSAA	A224T	pCT26-13
C8	*Capn12*	VTGTHKMSLGFTK**A**RLLRLRNPWGRVE	V276A	
C9	*Kdelr1*	LWTFSIYLESVAI**M**PQLFMVSKTGEAE	L132M	
C10	*Aldh18a1*	LHSGQNHLKEMAI**S**VLEARACAAAGQS	P154S	
C11	*Anapc1*	GSLFGSSRVQYVV**N**PAVKIVFLNIDPS	D241N	
C12	*Pcbp4*	PPPGLAAYTAKMA**T**ANGSKKAERQKFS	A388T	
C13	*Nup205*	VCNVKLLHRVLVA**D**VNALQGMAAIGQR	E1239D	

WT: wildtype amino acid, AA#: position of mutated amino acid, Mut: mutated amino acid.

Supplementary Table 2. Overview of B16F10 neoepitopes used for *in vivo* studies and *ex vivo* analyses. Bold amino acid denotes the position of the point mutation(s). Blue amino acids show the best predicted MHC-I neoepitope. Underlined amino acids show the best predicted MHC-II neoepitope.

ID	Gene	AA sequence used for immunization	Substitution (WT, AA#, Mut)	DNA plasmids
B1	*Pcmtd1*	KNILAVSFAPLVQ**L**SKNDNGTPDSVGL	P222L	pB16F10-13
B2	*Dennd5a*	LLADCPITAHMYE**A**VALIK**G**HTLVNSL	D1250A, D1256G	
B3	*Dhx37*	DPVFIHPSSVLFK**G**LPEFVVYQEIVET	E968G	
B4	*Gpatch4*	QAFLAQLKGSKAL**A**TSQPLTDSEPSQK	G192A	
B5	*Rpl13a*	GRGHLLGRLAAIV**G**KQVLLGRKVVVVR	A24G	
B6	*Mta1*	LEAVLRYLETHPR**L**PKPDPVKSSSSVL	P530L	
B7	*Nr1h2*	ELCRVCGDKASGF**R**YNVLSCEGCKGFF	H89R	
B8	*Ndufs6*	MAA**A**LTFRRLLTLPRAA	V4A	
B9	*Snx5*	ELINFKRKRVAAF**Q**KNLIEMSELEIKH	R373Q	
B10	*Grpel1*	LEEQLRETMEKYK**C**ALADTENLRQRSQ	R80C	
B11	*Ctsd*	SYYTVFDRDNNRV**S**FANAVVL	G403S	
B12	*Golgb1*	QRAAAPSAASSPA**D**VQSLKKAMSSLQN	E2814D	
B13	*Cd2ap*	FNGGHSPTQSPEK**I**LKLPKEDDSGNLK	T518I	
M27	*Obsl1*	REGVELCPGNKYE**M**RRHGTTHSLVIHD	T1764M	pB16F10-ctrl.
M30	*Kif18b*	PSKPSFQEFVDWE**N**VSPELNSTDQPFL	K739N	
M33	*Pbk*	DSGSPFPAAVILR**D**ALHMARGLKYLHQ	V145D	
Trp2	*DCT*	SVYDFFVWL	NA	

WT: wildtype amino acid, AA#: position of mutated amino acid, Mut: mutated amino acid.
