# Supplementary material for: DNA based neoepitope vaccination induces tumor control in syngeneic mouse models
Source: NPJ Vaccines. 2023 May 27;8:77. doi: 10.1038/s41541-023-00671-5 (PMC10224666; doi:10.1038/s41541-023-00671-5)
Supplement: Supplementary file 2 — REPORTING SUMMARY [file 41541_2023_671_MOESM2_ESM.pdf]

nature portfolio

Corresponding author(s): DBPR NPJVACCINES-02531-T

Last updated by author(s): Jan 25, 2023

Reporting Summary

Nature Portfolio wishes to improve the reproducibility of the work that we publish. This form provides structure for consistency and transparency in reporting. For further information on Nature Portfolio policies, see our Editorial Policies and the Editorial Policy Checklist.

Statistics

For all statistical analyses, confirm that the following items are present in the figure legend, table legend, main text, or Methods section.

n/a	Confirmed	
☐	☒	The exact sample size (*n*) for each experimental group/condition, given as a discrete number and unit of measurement
☐	☒	A statement on whether measurements were taken from distinct samples or whether the same sample was measured repeatedly
☐	☒	The statistical test(s) used AND whether they are one- or two-sided<br>*Only common tests should be described solely by name; describe more complex techniques in the Methods section.*
☒	☐	A description of all covariates tested
☐	☒	A description of any assumptions or corrections, such as tests of normality and adjustment for multiple comparisons
☒	☐	A full description of the statistical parameters including central tendency (e.g. means) or other basic estimates (e.g. regression coefficient) AND variation (e.g. standard deviation) or associated estimates of uncertainty (e.g. confidence intervals)
☐	☒	For null hypothesis testing, the test statistic (e.g. *F*, *t*, *r*) with confidence intervals, effect sizes, degrees of freedom and *P* value noted<br>*Give P values as exact values whenever suitable.*
☒	☐	For Bayesian analysis, information on the choice of priors and Markov chain Monte Carlo settings
☒	☐	For hierarchical and complex designs, identification of the appropriate level for tests and full reporting of outcomes
☒	☐	Estimates of effect sizes (e.g. Cohen's *d*, Pearson's *r*), indicating how they were calculated

Our web collection on statistics for biologists contains articles on many of the points above.

Software and code

Policy information about availability of computer code

Data collection	Graphpad Prism version 9 for Mac OS X
Data analysis	Graphpad Prism version 9 for Mac OS X and FlowJo software (version 10.8.0) for Mac OS X<br><br>Python v2.7.15; NetMHCIIpan v3.1; Cutadapt v1.18; BWA v0.7.12; samtools v1.9; DeepVariant v0.10.0; LoFreq v2.1.4; GATK (Mutect2) v 4.0.8.1; Strelka v2.7.1; SNVSniffer v2.0.4; Sequenza v3.0.0; STAR v2.5.2b; RSEM v1.2.29

For manuscripts utilizing custom algorithms or software that are central to the research but not yet described in published literature, software must be made available to editors and reviewers. We strongly encourage code deposition in a community repository (e.g. GitHub). See the Nature Portfolio guidelines for submitting code & software for further information.

Data

Policy information about availability of data

All manuscripts must include a data availability statement. This statement should provide the following information, where applicable:

- Accession codes, unique identifiers, or web links for publicly available datasets
- A description of any restrictions on data availability
- For clinical datasets or third party data, please ensure that the statement adheres to our policy

The datasets generated during and/or analysed during the current study are available from the corresponding author on reasonable request.

Human research participants

Policy information about studies involving human research participants and Sex and Gender in Research.

Reporting on sex and gender	n/a
Population characteristics	n/a
Recruitment	n/a
Ethics oversight	n/a

Note that full information on the approval of the study protocol must also be provided in the manuscript.

Field-specific reporting

Please select the one below that is the best fit for your research. If you are not sure, read the appropriate sections before making your selection.

☒ Life sciences ☐ Behavioural & social sciences ☐ Ecological, evolutionary & environmental sciences

For a reference copy of the document with all sections, see nature.com/documents/nr-reporting-summary-flat.pdf

Life sciences study design

All studies must disclose on these points even when the disclosure is negative.

Sample size	As described by Dell et al. (2002, ILAR J) sample size was determined by the formula for continuous variables in studies comparing two group means: n = 1 + 2C (s/d)^2, where s: standard deviation of the variable, d: the magnitude of the difference, and C: depends on the power and significance level.
Data exclusions	No data was excluded
Replication	All data shown are from independent immunization experiments. Replicates is stated for each experiment and assay.
Randomization	For all studies mice were housed in mixed cages, so all groups and treatment regimens were distributed across cages. For in vivo studies with checkpoint inhibitor therapy mice were randomized into groups based on tumor size one day prior to initiation of checkpoint inhibitor therapy. For all other in vivo studies mice were placed into treatment groups wihtout randomization, albeit still with mixed cages/group distribution.
Blinding	n/a

Reporting for specific materials, systems and methods

We require information from authors about some types of materials, experimental systems and methods used in many studies. Here, indicate whether each material, system or method listed is relevant to your study. If you are not sure if a list item applies to your research, read the appropriate section before selecting a response.

Materials & experimental systems

n/a	Involved in the study	
☐	☒	Antibodies
☐	☒	Eukaryotic cell lines
☒	☐	Palaeontology and archaeology
☐	☒	Animals and other organisms
☒	☐	Clinical data
☒	☐	Dual use research of concern

Methods

n/a	Involved in the study	
☒	☐	ChIP-seq
☐	☒	Flow cytometry
☒	☐	MRI-based neuroimaging

Antibodies

Antibodies used	All antibodies used (and cat. numbers) are stated in the materials and methods section of the manuscript.
Validation	All antibodies were used according to manufacturer's instructions or titrated to determine optimal staining concentration on relevant material with known positive signals.

Eukaryotic cell lines

Policy information about cell lines and Sex and Gender in Research

Cell line source(s)	All cell lines used are of Mus musculus origin and were purchased from ATCC, B16F10 (cat. CRL-6475), CT26 (cat. CRL-2638). B16F10 cells are from a female mouse (Castle et al. 2012). There is no description of the sex of the CT26 cell line.
Authentication	Both cell lines were subjected to NGS sequencing. Furthermire cells were authenticated via observation of morphology during in vitro expansion and B16F10 cells were confirmed to be dark due to melanin production, as described by vendor.
Mycoplasma contamination	Cell lines were tested negative for mycoplasma.
Commonly misidentified lines (See ICLAC register)	N/A

Animals and other research organisms

Policy information about studies involving animals; ARRIVE guidelines recommended for reporting animal research, and Sex and Gender in Research

Laboratory animals	6-8 week old BALB/c JrJ and C57BL/6 JrJ female mice were acquired from Janvier Labs (France).
Wild animals	The study did not invovle wild animals.
Reporting on sex	Only female mice were used in this study.
Field-collected samples	The study did not invovle field-collected samples.
Ethics oversight	The experiments were conducted under license 2017-15-0201-01209 from the Danish Animal Experimentation Inspectorate in accordance with the Danish Animal Experimentation Act (BEK nr. 12 of 7/01/2016), which is compliant with the European directive (2010/63/EU).

Note that full information on the approval of the study protocol must also be provided in the manuscript.

Flow Cytometry

Plots

Confirm that:

☒ The axis labels state the marker and fluorochrome used (e.g. CD4-FITC).

☒ The axis scales are clearly visible. Include numbers along axes only for bottom left plot of group (a 'group' is an analysis of identical markers).

☒ All plots are contour plots with outliers or pseudocolor plots.

☒ A numerical value for number of cells or percentage (with statistics) is provided.

Methodology

Sample preparation	Blood: tail vein blood was collected in EDTA-coated tubes (Sarstedt, #20.1278.100) and then transferred to deep, 96-well plates (Sigma, #575653). After relevant staining blood underwent one-step fixation/red blood cell lysis (eBioscience

#00-5333-57) before acquisition.

Spleens: spleens were collected in cold RPMI supplemented with 10% FCS, followed by processing to single cells suspensions via GentleMACS processing (Miltenyi Biotec, C-tubes #130-096-334 and Dissociater #130-093-235) and passage through a 70 mm filter (Corning, CLS431751). Splenocytes were cryopreserved in FCS with 10% DMSO (Merck, #D8418).

Tumors: Isolated tumors were dissociated into single cell suspensions with a cocktail of tumor dissociation enzymes (Miltenyi Biotech #130-096-730) and filtered through 70-µm cell strainers according to the instructions of the manufacturer. Tumor single cell suspensions were cryopreserved in FCS with 10% DMSO (Merck, #D8418).

Spleens and tumors were thawed with warm RPMI supplemented with 10% FCS, washed and counted before further applications, staining and acquisition.

Instrument	Flow cytometer: BD FACS Celesta Cell Analyzer (BVYG configuration)
Software	FACS acquisition in BD FACS Diva, FACS gating and analysis via FlowJo software (version 10.8.0).
Cell population abundance	Cells were not FACS sorted. Cell populations are described in the manuscript and representative examples are visible in the supplementary figures (gating strategies).
Gating strategy	Gating strategies varied from assay to assay and representative examples are shown in supplementary figures.

☒ Tick this box to confirm that a figure exemplifying the gating strategy is provided in the Supplementary Information.
